# *Tet1* Isoforms Differentially Regulate Gene Expression, Synaptic Transmission, and Memory in the Mammalian Brain

**DOI:** 10.1523/JNEUROSCI.1821-20.2020

**Published:** 2021-01-27

**Authors:** C.B. Greer, J. Wright, J.D. Weiss, R.M. Lazarenko, S.P. Moran, J. Zhu, K.S. Chronister, A.Y. Jin, A.J. Kennedy, J.D. Sweatt, G.A. Kaas

**Affiliations:** ^1^Department of Pharmacology, Vanderbilt University, Nashville, Tennessee 37232; ^2^Department of Chemistry, Bates College, Lewiston, Maine 04240

**Keywords:** epigenetics, gene expression, memory, neurons, synaptic transmission, TET1

## Abstract

The dynamic regulation of DNA methylation in postmitotic neurons is necessary for memory formation and other adaptive behaviors. Ten-eleven translocation 1 (TET1) plays a part in these processes by oxidizing 5-methylcytosine (5mC) to 5-hydroxymethylcytosine (5hmC), thereby initiating active DNA demethylation. However, attempts to pinpoint its exact role in the nervous system have been hindered by contradictory findings, perhaps due in part, to a recent discovery that two isoforms of the *Tet1* gene are differentially expressed from early development into adulthood. Here, we demonstrate that both the shorter transcript (*Tet1^S^*) encoding an N-terminally truncated TET1 protein and a full-length *Tet1* (*Tet1^FL^*) transcript encoding canonical TET1 are co-expressed in the adult mouse brain. We show that *Tet1^S^* is the predominantly expressed isoform and is highly enriched in neurons, whereas *Tet1^FL^* is generally expressed at lower levels and more abundant in glia, suggesting their roles are at least partially cell type-specific. Using viral-mediated, isoform and neuron-specific molecular tools, we find that the individual repression of each transcript leads to the dysregulation of unique gene ensembles and contrasting changes in basal synaptic transmission. In addition, *Tet1^S^* repression enhances, while *Tet1^FL^* impairs, hippocampal-dependent memory in male mice. Together, our findings demonstrate that each *Tet1* isoform serves a distinct role in the mammalian brain.

**SIGNIFICANCE STATEMENT** In the brain, activity-dependent changes in gene expression are required for the formation of long-term memories. DNA methylation plays an essential role in orchestrating these learning-induced transcriptional programs by influencing chromatin accessibility and transcription factor binding. Once thought of as a stable epigenetic mark, DNA methylation is now known to be impermanent and dynamically regulated, driving neuroplasticity in the brain. We found that *Tet1*, a member of the ten-eleven translocation (TET) family of enzymes that mediates removal of DNA methyl marks, is expressed as two separate isoforms in the adult mouse brain and that each differentially regulates gene expression, synaptic transmission and memory formation. Together, our findings demonstrate that each *Tet1* isoform serves a distinct role in the CNS.

## Introduction

DNA methylation is an essential regulator of gene expression in the brain, and is required for learning and memory formation (Jarome and Lubin, 2014). Based on its role during development, DNA methylation was initially thought to function as a stable epigenetic mark in postmitotic brain cells, but it is now known to be dynamically regulated, in response to neuronal stimulation, learning, and experience (Martinowich et al., 2003; Miller and Sweatt, 2007; Saunderson et al., 2016). DNA methylation levels are controlled by the antagonistic actions of DNA methyltransferases (DNMTs), which methylate the fifth carbon of cytosine bases [5-methylcytosine (5mC); Okano et al., 1999; Hermann et al., 2004], and the ten-eleven translocation (TET) enzymes, which oxidize 5mCs to 5-hydroxymethylcytosine (5hmC) and initiate active DNA demethylation (Tahiliani et al., 2009; Guo et al., 2011). TET enzymes are critical for brain function and mutations or changes in the expression of *Tet* genes are associated with, or the cause of, cognitive deficits in humans (Dong et al., 2015; Cochran et al., 2020; Beck et al., 2020). Thus, the study of TET-mediated mechanisms in the brain may provide novel insights into the pathophysiology of neurologic disease.

All three *Tet* genes (*Tet1-3*) are expressed in the mammalian brain and studies suggest they generally serve non-redundant functions. *Tet3* is the highest expressed and is transcriptionally upregulated by neuronal stimulation (Widagdo et al., 2014). Knock-down (KD) of *Tet3* alters synaptic transmission, and conditional knock-out (KO) of the gene impairs spatial memory, indicating that its necessary for cognition (Yu et al., 2015; Antunes et al., 2020). *Tet2* is also abundantly expressed in the brain and its disruption is associated with enhanced spatial memory, suggesting it may function as a negative regulator of neuroplasticity (Zengeler et al., 2019). *Tet1*, despite much lower expression, has been the most studied *Tet* family member in the nervous system, implicated in the regulation of activity-dependent gene expression, synaptic transmission, and cognition (Alaghband et al., 2016). However, attempts to define its exact role, particularly in the context of learning and memory, have been hampered by contradictory findings. For instance, depending on the study, loss of *Tet1* in KO mice has been reported to either impair, enhance, or have no effect at all on memory (Rudenko et al., 2013; Zhang et al., 2013; Kumar et al., 2015; Towers et al., 2018). Likewise, overexpression of *Tet1* has been shown to enhance memory, while expression of its catalytic domain does the opposite (Kaas et al., 2013; Kwon et al., 2018). One potential explanation for these inconsistences comes from a recent report that the *Tet1* gene undergoes an isoform switch from the full-length, canonical transcript (hereafter *Tet1^FL^*) in embryonic stem cells to a shorter, truncated variant (hereafter *Tet1^S^*) exclusively expressed to somatic tissues (Zhang et al., 2016). In addition, evidence suggests that in some tissues both transcripts might be co-expressed (Good et al., 2017; Yosefzon et al., 2017). Whether this is the case in the adult brain, and if so, what functions these *Tet1* isoforms might serve, has not been explored.

Here, we report that both *Tet1* isoforms are expressed in the brain. The *Tet1^S^* isoform is highly enriched in neurons and its expression is regulated in an activity-dependent manner. In contrast, *Tet1^FL^* is transcribed at low basal levels in neurons, yet is much more abundant in glia, suggesting the functions of each isoform may be at least partially cell-type specific. Using newly-developed molecular tools, we found that the transcriptional repression of each individual isoform results in distinct changes in neuronal gene expression, basal synaptic transmission and memory formation, demonstrating that *Tet1^FL^* and *Tet1^S^* serve important, non-redundant functions in the nervous system.

## Materials and Methods

### 

#### Animals

Experiments were performed using two- to three-month-old C57BL/6J male mice originally purchased from The Jackson Laboratory. All mice were group housed, kept under 12:12 light/dark cycles, with food and water available *ad libitum*. For stereotaxic surgeries and behavioral assays, four-week-old male littermates were purchased, housed in sets of three to five animals and aged out to 10 weeks before experimentation in an effort to minimize fighting. All procedures and behavioral assays were approved by the Vanderbilt Animal Care and Use Committee.

#### Chromatin immunoprecipitation (ChIP)

Tissue samples were cut into ∼1-mm pieces using a razor and incubated in a 1× PBS solution containing 1% formaldehyde and proteinase inhibitors for 10 min at 37°C, followed by the addition of glycine (final concentration 125 mm) to quench the reaction. Samples were washed 6× with ice-cold PBS and then homogenized in lysis buffer (50 mm Tris, pH 8.1, 10 mm EDTA, and 1% SDS) using a pestle. Chromatin was sheared using a Bioruptor Pico set to three cycles of 30 s on, 30 s off. Samples were then processed using the Magna ChIP G Tissue kit (EMD Millipore) according to manufacturer instructions. Briefly, samples were precleared using 25 μl of Protein G beads, then placed on a rotator and incubated overnight at 4°C with 25 μl of Protein G beads and 4 μl (1 mg/ml) of anti-RNA polymerase II antibody (102660; Active Motif). Immune complexes were sequentially washed according to kit instructions. To reverse cross-links, samples were incubated at 65°C for 2 h in the presence of SDS and Proteinase K, then at 95°C for 10 min. Enriched DNA samples were purified with a QIAGEN PCR clean up kit. Purified DNA was then stored at −20°C or used immediately for quantitative real-time PCR (qRT-PCR). Fold enrichment for each primer set was normalized to input and reported relative to a negative control gene desert region (mouse Igx1a locus; QIAGEN). Primers: *Tet1^FL^* promoter F 5′-gcactctgcaactggtttg-3′, R 5′-gtagaagaggcaggtagaggta-3′; *Tet1^S^* promoter F 5′-ctgctttgaaacaccatgataa-3′, R 5′-tagccatcttgcctgctt-3′.

#### 5′ rapid amplification of cDNA ends (RACE)

Total RNA was extracted from adult mouse hippocampal tissue using an RNeasy Plus kit (QIAGEN). Amplification of 5′ cDNA ends was performed using the GeneRacer kit (Life Technologies) in accordance with manufacturer instructions. Briefly, 3-μg total RNA was dephosphorylated, decapped, and then ligated to a GeneRacer 5′ RNA oligo. Between each step, samples were purified by phenol:chloroform extraction and precipitated with ethanol and glycogen. cDNA was then synthesized from the ligated RNA using Superscript III reverse transcriptase (Invitrogen) and random primers. cDNA samples were amplified for two rounds by nested PCR using the Platinum PCR Supermix High Fidelity (Invitrogen) kit and 5′ GeneRacer forward and reverse *Tet1-*isoform specific primers (*Tet1^FL^* 5′ RACE outside, 5′-ttgggtgtgactactgggcgctgggaga-3′; *Tet1^FL^* RACE nested, 5′-ggcgctgggagagtcgccagctaaga-3′; *Tet1^S^* 5′ RACE outside, 5′-agccaggcttctggaagagcagggtgt-3′; *Tet1^S^* RACE nested, 5′-cccggaggtggtgacactcatggcatcctt-3′). Purified PCR products were then cloned into pCR-Blunt II-TOPO vectors (Invitrogen) and subjected to sanger sequencing (GenHunter Corp.).

#### qRT-PCR

Total RNA was extracted from samples using an RNeasy Plus Mini kit (QIAGEN) and eluted in 30–50 μl of RNase free water. Sample concentration and purity were analyzed using a NanoDrop One Microvolume UV-Vis Spectrophotometer (Thermo Scientific) and cDNA synthesis was conducted in 20-μl reactions using the iScript cDNA Synthesis kit (Bio-Rad), or 10-μl reaction using SuperScript VILO master mix (Invitrogen) according to manufacturer instructions. All cDNA reactions were diluted 1:5 with RNase-free water to reduce the influence of any PCR inhibitors. qRT-PCR was performed on an CFX96 RT-PCR detection system in 10-μl reactions containing SsoAdvanced Universal SYBR Green Supermix and 200–300 μm primer and 1 μl of cDNA. All qRT-PCR primers were either designed using Primer Quest (Integrated DNA Technologies) to span exon-exon junctions or were acquired directly as predesigned PrimeTime qPCR Primer Assays (Integrated DNA Technologies). Relative fold quantification of gene expression between samples was calculated using the comparative C_t_ method (Livak and Schmittgen, 2001)and normalized to the geometric mean of three reference genes *Hypoxanthine phosphoribosyltransferase* (*Hprt*), *Glyceraldehyde-3-Phosphate Dehydrogenase* (*Gapdh*), and *Glucuronidase Beta* (*Gusb*). IDT PrimeTime qPCR Probe Assays: *Hprt*, Mm.PT.39a.22214828; *Gapdh*, Mm.PT.39a.1. Primers: *Gusb*, F 5′-cagactcagttgttgtcacct-3′, R 5′-tcaacttcaggttcccagtg-3′; *Tet1^FL^* F 5′-ctccctggtcatgtacctcta-3′, R 5′-gtaagtaaagatgcaaggatgcg-3′; *Tet1^S^* F 5′-cctccatctttatttatgcaag-3′, R 5′-ggtttgttgttaaagtctgtct-3′; *Neuronal PAS domain protein 4* (*Npas4*) F 5′-cacgtcttgatgacaatatgcc-3′, R 5′-ccaagttcaagacagcttcca-3′; *Activity regulated cytoskeleton associated protein* (*Arc*) F 5′-acgatctggcttcctcattctgct-3′, R 5′-aggttccctcagcatctctgcttt-3′; *Early growth response 1* (*Egr1*) F 5′-agcgccttcaatcctcaag-3′, R 5′-tttggctgggataactcgtc-3′.

#### Mouse primary neuron, glia and neuroblastoma 2a cultures

Mouse hippocampi from C57BL/6J P0 pups were dissected in ice-cold HBSS (Invitrogen) and digested with papain (Worthington) for 25 min at 37°C. Samples were then washed 2× in HBSS and dissociated by pipetting up and down 15–20 times through a P1000 pipette in growth media [Neurobasal media supplemented with 5% fetal bovine serum (FBS), 500 nm L-glutamine, 1× B27 (Invitrogen), and Penicillin-Streptomycin (Pen-Strep)]. The cell suspension was passed through a 100 μm filter and centrifuged for 5 min at 500 × *g*. Cell pellets were resuspended in growth media and seeded on poly-d-lysine coated (Sigma) 12-well plates (Corning) at ∼250–300 × 10^3^ cells per well or on 24-well plates at 100 × 10^3^ cells per well; 24 h later, media was replaced with maintenance media (Neurobasal media supplemented with 500 nm L-glutamine, 1 × B27) without Pen-Strep. At Days in vitro (DIV) 3–4, 1 μm 5-fluorodeoxyuridine (FdU) was added to the media for 24 h, then removed, to inhibit mitotic cell growth (Hui et al., 2016). Primary mixed glial cultures were prepared identically to neurons, except they were plated, then maintained, using media that consisted of 1× DMEM (+4.5 g/l D-glucose and L-glutamine, Invitrogen), Pen-Strep and 10% FBS. DIV10–DIV14 neuronal and glial cultures were used for qRT-PCR experiments. GSK126 or GSK343 were dissolved in Dimethyl sulfoxide (DMSO), and applied to cells for 72 h. Stimulation of neurons was conducted by adding 25 μl of maintenance media alone (vehicle), or media containing KCl, bicuculline, and NMDA/glycine (gly; final concentrations 25 mm, 50 μm, 10 μm/2 μm, respectively). Drugs were administered to each well and incubated for 1, 2, or 4 h. Mouse neuroblastoma 2a (N2a) cells were purchased from ATCC, and cultured using the same media as glial cells. For transfection experiments, N2a cells were seeded in 24-well plates at a density of 200 × 10^3^ per well and transfected using GenJet Reagent (II) in accordance with manufacturer instructions.

#### Transcription activator like effector (TALE) vector construction and analysis

All constructs used in this study were generated using Gibson Assembly methodology as previously described (Gibson et al., 2009). Briefly, primers were designed using NEBuilder Assembly Tool (v1.12.18) to generate overlapping (20–25 bp) PCR fragments amplified using Q5 High-Fidelity DNA Polymerase (NEB). Modified TALE constructs were created using PCR fragments amplified from pAAV-CW3SL-EGFP (Addgene #61463), a gift from Bong-Kiun Kaang (Choi et al., 2014), and constructs contained in the TALE Toolbox (Addgene kit #1000000019), a gift from Feng Zhang. TALE DNA targeting sequences were assembled into TALE construct backbones using Golden Gate Assembly Cloning, as previously described (Sanjana et al., 2012). TALE sequences targeting the *Tet1^FL^* (5′-TGCCCCAGCTACACTCCT-3′, sense) and *Tet1^S^* (5′-TCGCAGCCTAGCACTATC-3′, antisense) promoter regions were designed using TAL Effector Nucleotide Targeter 2.0 software (Doyle et al., 2012). Potential off-target genes for each sequence were identified using a UNIX shell script relying on bowtie (Langmead et al., 2009) with option –a to report all alignments and –v three to find sites with up to three mismatches in the mouse genome (mm10). The output was converted with samtools (Li et al., 2009), and homer annotatePeaks.pl (Heinz et al., 2010) was used to annotate the location of mismatched sequences relative to annotated genes and to determine the proximity to the promoter. Genes previously identified as TET1 targets in ChIP sequencing (ChIP-seq) datasets were excluded (Lachmann et al., 2010).

#### Immunostaining

For immunocytochemistry, cells were plated on glass cover slips coated with poly-d-lysine. Cells were washed 2× with ice cold 1× PBS, followed by fixation with fresh 4% paraformaldehyde for 15 min at room temperature (RT), blocked for 1 h at RT (10% goat serum and 0.3% Triton X-100 in 1× PBS), and incubated with primary antibodies (anti-HA, ab18181; anti-GFP, ab13970; anti-NeuN, ab104224; Abcam) at a concentration of 1:1000 at RT for 2 h or overnight at 4°C in 1:3 diluted blocking buffer. Slides were then washed and incubated with the appropriate Alexa Flour secondary antibodies (Abcam) at a concentration of 1:1000 for 1 h at RT. Slides were mounted using ProLong Gold Antifade Mountant with DAPI (Invitrogen) and images were acquired using a IX73 microscope (Olympus) and cellSens standard software.

#### AAV generation and viral injections

High titers (>10^13^ genome copies/ml) of AAV1 viral particles containing the *Tet1* isoform TALE constructs were packaged by Applied Biological Materials (ABM). For primary hippocampal neuron experiments in 12-well or 24-well plates, we added 1 μl of AAVs diluted 1:10. For *in vivo* experiments involving stereotaxic surgeries, AAVs were injected bilaterally into the dorsal hippocampus (dHPC) of 10-week-old mice using the following stereotaxic coordinates: –2.0 mm AP, ±1.5 mm ML, and −1.6 mm DV from bregma. A total of 1.5 μl of viral solution was injected per hemisphere. Injections were performed using a 10-ml Hamilton Gastight syringe controlled by a Pump 11 Elite Nanomite Programmable Syringe Pump (Harvard Apparatus). Injections proceeded at a speed of 150 *n*l min^−1^ through a 32-gauge needle. The injection needle was left in place an additional 5 min. Behavior and gene expression experiments involving AAV delivery *in vivo*, were either begun or performed 14 days after surgery, respectively.

#### Behavior

All behavioral assays were conducted in the Vanderbilt Mouse Neurobehavioral Core (https://lab.vanderbilt.edu/mouse-core/) by blinded experimenters.

##### Elevated zero maze (EZM)

Mice were placed on the open section of the maze (White 2325–0229, San Diego Instruments) and allowed to explore freely for 5 min. Video recording and tracking were performed using ANY-maze video tracking software (Stoelting Co).

##### Open field

Mice were placed in the center of a large Plexiglas box (43 × 42 × 30 cm), and locomotor activity was measured for 30 min (Med Associates). Data are presented as total distance traveled in centimeters.

##### Contextual fear conditioning (CxFC)

Fear conditioned mice used for behavioral analysis were trained in a novel context (catalog #MED-VFC2-SCT-M, Med Associates Inc.) using a 3.5-min training protocol consisting of a 3-min habituation period, followed by a single foot shock (0.5 mA, 2 s). Mice were removed from the chamber 30 s later. To assess long-term memory formation, mice were placed back in the same context for 5 min in the absence of the unconditioned stimulus. Percent freezing was calculated automatically using Video Freeze version 2.1.0 (Med Associates Inc.). Fear conditioned animals used for gene expression analysis were trained using a protocol consisting of three context-shock pairings as described above (0.75 mA, 2 s) every 2 min, and removed from the apparatus after 7 min. At the conclusion of behavioral testing, hippocampi were removed from all animals and AAV1-mediated enhanced green fluorescent protein (EGFP) expression was examined using an IX73 microscope (Olympus). Because of the reported role of TET1 in neurogenesis (Zhang et al., 2013), any animals displaying EGFP expression in the dentate gyrus were excluded from our study.

#### RNA-seq and ChIP-seq

For RNA-seq, total RNA was extracted from AAV1-transduced primary hippocampal neurons (DIV12–DIV14) using an AllPrep DNA/RNA/Protein Mini kit (QIAGEN). Total RNA was poly A selected and sequenced (Hudson Alpha GSL) on the Illumina platform (HiSeq v.4, paired end, 50 bp, 50 million reads). Reads were passed through a quality filter with trimmomatic (Bolger et al., 2014) using recommended settings for paired end libraries, and adaptor sequences matching TruSeq3_PE were trimmed. Surviving reads were aligned to the mm10 genome with hisat2 (Kim et al., 2015). Stringtie (Pertea et al., 2015) was used to incorporate any novel transcripts from these sequencing libraries into the mm10 Refseq annotation of known transcripts, and featurecounts (Liao et al., 2014) attributed reads to the resulting custom annotation. EdgeR (Robinson et al., 2010; McCarthy et al., 2012) was used for determining fold change and false discovery rates (FDRs) for each gene with sufficient read depth. Statistics in EdgeR were determined with genewise negative binomial generalized linear models with quasi-likelihood tests (glmQLFit function). H3K4me3 ChIP-seq from CA1 neurons was downloaded from (Halder et al., 2016). Alignment, peak calling, and file visualization were conducted as described previously (Collins et al., 2019). RNA-seq datasets generated in this study have been deposited in Gene Expression Omnibus (GEO) with the accession number GSE140174.

#### Electrophysiology

Whole-cell voltage clamp recordings were performed on neurons from 14 to 21 DIV mouse hippocampal cultures using a Multi-Clamp 700B amplifier. Signals were digitized through a Digidata 1440A at 20 kHz, filtered at 1.8 kHz, and analyzed offline with Clampfit 10.7 software (Molecular Devices). Cells were held at −60 mV. Patch pipettes were pulled from borosilicate glass capillaries with resistances ranging from 3 to 6 MΩ when filled with pipette solution, containing the following:120 mM cesium methanesulfonate, 5 mM CsCl, 1 mM MgCl_2_, 1 mM CaCl_2_, 10 mM HEPES, 2 mM EGTA, 4 mM Na-ATP, 0.4 mM Na-GTP, 10 mM phosphocreatine, 3 mM Na-ascorbate, and 5 mM glucose, pH 7.2. The bath solution (Tyrode's saline) contained the following:150 mM NaCl, 4 mM KCl, 2 mM MgCl_2_, 2 mM CaCl_2_, 10 mM HEPES, and 10 mM glucose, pH 7.35. For the recordings of miniature excitatory postsynaptic currents (mEPSCs), bath solution was supplied with 1 μm tetrodotoxin (TTX, Hello Bio). mEPSC events were collected from the recorded traces using a template-based approach. Templates were generated from traces recorded under control conditions. 30 randomly selected events from individual recordings were used for analysis.

#### Experimental design and statistical analyses

Statistical analysis and graphing of non-genomic data were performed using GraphPad prism 7.04. For two groups, statistical significance was determined using the Student's *t* test. For three or more groups, statistical significance was determined using one-way or two-way analysis of variance (ANOVA), followed by Dunnett's multiple comparisons tests *post hoc*. Statistical significance of cumulative distributions was determined using the Kolmogorov–Smirnov test.

## Results

### *Tet1* is expressed as two distinct transcripts in the adult brain

**Figure 1.**
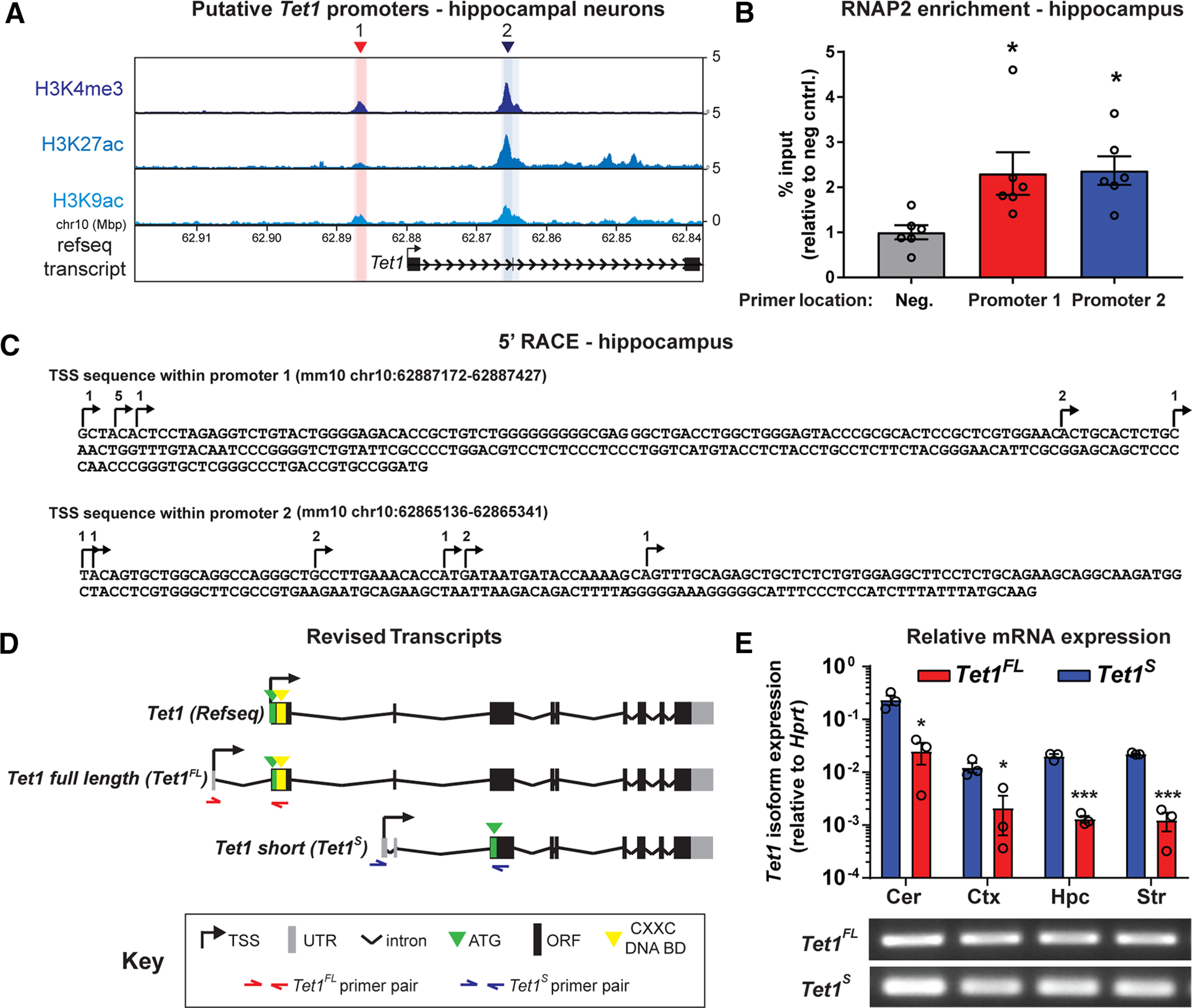
*Tet1* is expressed as two distinct transcript isoforms in the adult mouse brain. ***A***, Mean normalized H3K4me3, H3K27ac and H3K9ac ChIP-seq signal in NeuN+ hippocampal neurons at the *Tet1* gene locus (Halder et al., 2016). Red and blue shaded areas depict putative *Tet1* isoform promoter regions relative to the annotated RefSeq *Tet1* transcript. Chr10 = Chromosome 10, Mbp = Mega base pairs. ***B***, ChIP-qRT-PCR analysis of RNAP2 enrichment (% input) at predicted *Tet1* promoters in adult hippocampal tissue relative to a negative control (mouse Igx1a); *p < 0.05 (Dunnett's *post hoc* test), *p* < 0.05 (one-way ANOVA); *n* = 6 mice. Data represent mean ± SEM. ***C***, 5′ RACE sequence results summary from DNA clones amplified from adult hippocampal RNA; *n* = 8–10 clones/isoform. Arrows and numbers above each nucleotide represent the TSSs and number of clones, respectively. ***D***, Illustration of revised *Tet1* isoform transcript architecture based on ChIP and 5′ RACE data. Gray, untranslated region (UTR); black, Open Reading Frame (ORF); green, ATG (Translation Start Site); yellow, CXXC non-methyl CpG binding domain. Half arrows represent isoform specific primer locations. ***E***, top, qRT-PCR analysis of *Tet1* isoform expression levels in adult brain sub-regions relative to *Hprt*. Cer = cerebellum, Ctx = cortex, Hpc = hippocampus, Str = striatum. Bottom, Image of endpoint PCR products generated after qRT-PCR using *Tet1^FL^* and *Tet1^S^*-specific primers; *p < 0.05, ***p < 0.001 (unpaired two-tailed *t* test); *n* = 3 per group. Data represent mean ± SEM (Standard Error of the Mean).

In order to establish whether the *Tet1* gene is expressed as more than one transcript in the adult brain, we first examined the *Tet1* 5′ coding region for promoter-associated histone marks using published ChIP-seq datasets derived from adult NeuN+ hippocampal neurons (Halder et al., 2016). We found two regions within the *Tet1* gene locus that were enriched with H3 lysine four tri-methylation (H3K4me3), H3 lysine 27 acetylation (H3K27ac), and H3 lysine 9 acetylation (H3K9ac); marks typically associated with transcriptionally active promoters (Liang et al., 2004; Gates et al., 2017; Sato et al., 2019). The distal site, termed promoter 1, was located upstream of *Tet1* Refseq exon 1 and only mildly enriched for the three epigenetic modifications, whereas the second region, termed promoter 2, was located in an intronic region just upstream of *Tet1* Refseq exon 2 and was characterized by much stronger active histone peaks (Fig. 1*A*). To test whether like other active promoters, RNA polymerase II (RNAP2) was enriched at these sites, we conducted ChIP on hippocampal chromatin using primers targeted to each region. At both sites, we observed significant RNAP2 enrichment compared with a negative control region (fold change: *F*_(2,15)_ = 5.2, *p* = 0.0198; one-way ANOVA; negative control, 1 ± 0.16 vs site 1, 2.3 ± 0.5, *p* = 0.029, negative control, 1 ± 0.16 vs site 2, 2.4 ± 0.3, *p* = 0.023, *n* = 6 for all groups; Dunnett's *post hoc* test) indicating that each promoter was likely transcriptionally active (Fig. 1*B*).

Next, we used hippocampal RNA and 5′ RACE to examine whether the transcriptional start sites (TSSs) of any *Tet1* transcripts aligned to either of the predicted promoters. Our analysis identified two separate TSSs that corresponded to each promoter. As is common for many genes, their precise start sites varied by several nucleotides (Giardina and Lis, 1993; Leenen et al., 2016; Fig. 1*C*). The transcript starting at promoter 1, termed *Tet1 full length (Tet1^FL^)*, encodes the full length canonical TET1 enzyme translated from a start codon (Kozak sequence-gccATGt) located in exon 2 (Refseq *Tet1* exon 1). While the transcript arising from intronic promoter 2, termed *Tet1 shor*t (*Tet1^S^*), encodes for a truncated enzyme lacking a large portion of the TET1^FL^ N terminus, including the CXXC non-methylated CpG binding domain (Fig. 1*D*). TET1^S^ is translated from a start codon (Kozak sequence-tccATGg) located in the third exon of the Refseq annotated *Tet1* transcript.

In order to examine where, and to what extent, *Tet1^FL^* and *Tet1^S^* were expressed in the brain, we designed isoform-specific primers (Fig. 1*D*) to perform qRT-PCR using cDNA libraries generated from the cerebellum, cortex, hippocampus, and striatum. We detected both *Tet1* transcripts in all four brain regions, with the mRNA levels of *Tet1^S^* ∼10-fold higher than *Tet1^FL^* across all samples (Fig. 1*E*), indicating that it is the predominant *Tet1* transcript expressed in the brain. Notably, the levels of both transcripts were an order of magnitude higher in the cerebellum than any of other brain regions examined (*Tet1* isoform fold changes Cer: *S*, 0.23 ± 0.049 vs Cer: *FL*, 0.025 ± 0.011, *t*_(4)_ = 4.2, *p* = 0.014; Ctx: *S*, 0.016 ± 0.0026 vs Ctx: *FL*, 0.0021 ± 0.0015, *t*_(4)_ = 4.5, *p* = 0.011; Hpc: *S*, 0.02 ± 0.0018 vs Hpc: *FL*, 0.0013 ± 0.0002, *t*_(4)_ = 10, *p* = 0.0005; Str: *S*, 0.022 ± 0.00,057 vs Str: *FL*, 0.0013 ± 0.00,049, *t*_(4)_ = 28, *p* < 0.0001, *n* = 3 all groups; unpaired two-tailed *t* test). In addition, we measured *Tet1^FL^* and *Tet1^S^* mRNA levels in the adult heart, kidney, liver, muscle, and spleen. Both transcripts were present, and expressed at ratios comparable to those in the brain, suggesting this *Tet1* expression pattern is a general feature of most somatic tissues (data not shown). Taken together, our results demonstrate that two transcripts, encoding distinct protein isoforms, are actively generated from the *Tet1* gene in the adult mammalian brain and that the novel, truncated *Tet1^S^* is the predominant transcript.

### *Tet1* isoform transcript usage and regulation significantly differ between neurons and glia

**Figure 2.**
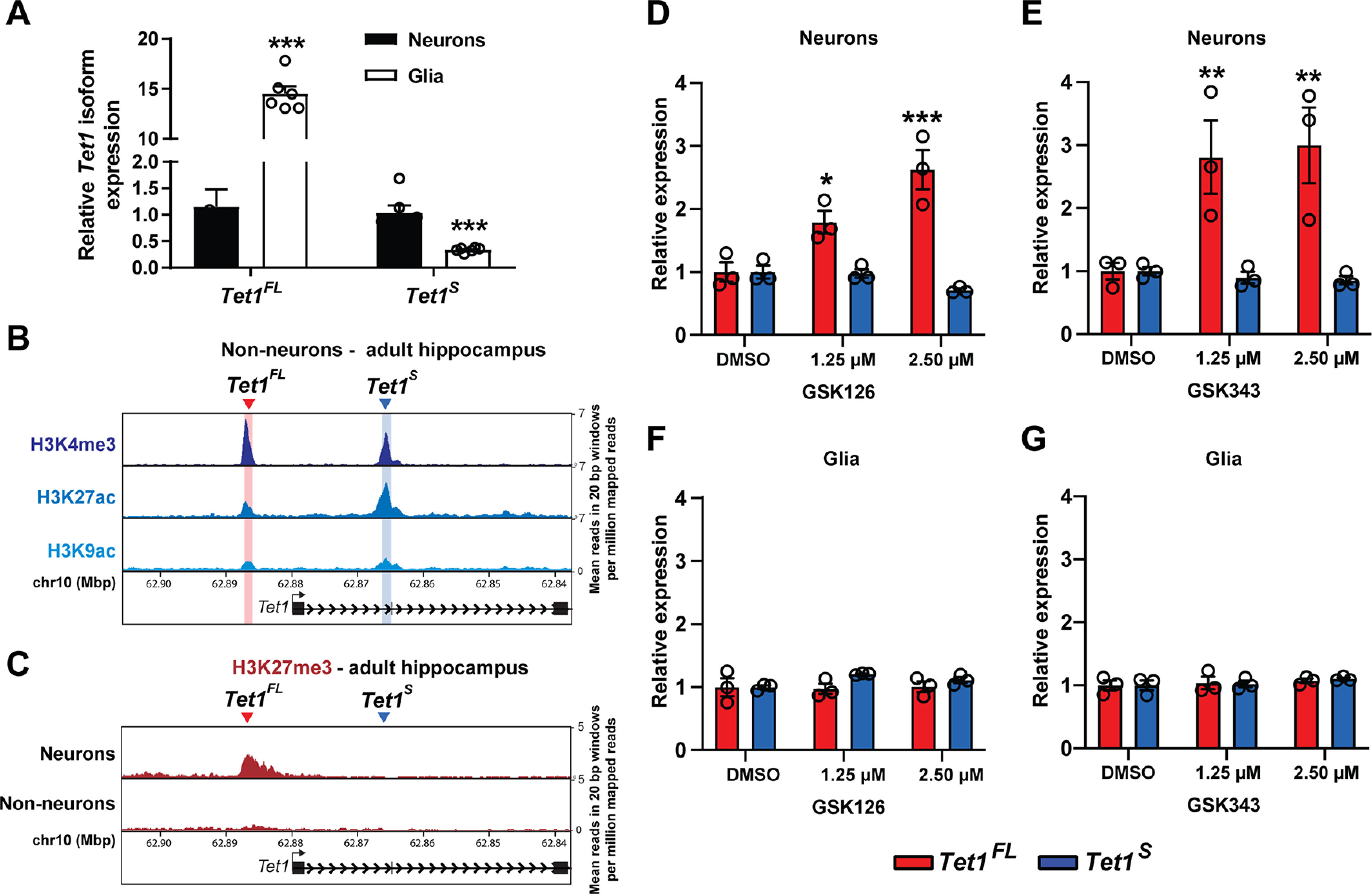
*Tet1* isoform transcript usage differs between neurons and non-neuronal cells in the brain. ***A***, qRT-PCR analysis of *Tet1^FL^* and *Tet1^S^* isoform expression levels in primary hippocampal glial cultures relative to hippocampal neuron cultures; ***p < 0.001 (unpaired two-tailed *t* test); *n* = 6. ***B***, Mean normalized H3K4me3, H3K27ac, and H3K9ac ChIP-seq signals in hippocampal non-neuronal cells (NeuN–) at the *Tet1* gene locus. ***C***, Comparison of mean normalized H3K27me3 ChIP-seq signals in hippocampal neurons (NeuN+) and non-neuronal (NeuN–) cells at the *Tet1* gene locus. Data for both ***B***, ***C*** generated from Halder et al. (2016). ***D***, ***E***, qRT-PCR analysis of *Tet1^FL^* and *Tet1^S^* isoform expression levels in primary hippocampal neurons treated with the EZH2 inhibitors GSK126 and GSK343, respectively. ***F***, ***G***, qRT-PCR analysis of *Tet1^FL^* and *Tet1^S^* isoform expression levels in primary hippocampal glia treated with the EZH2 inhibitors GSK126 and GSK343, respectively. Seventy-two-hour treatments of indicated doses were conducted. Data normalized to vehicle (DMSO) for each isoform; *p < 0.05, **p < 0.01, ***p < 0.001 (Dunnett's *post hoc* test vs vehicle); *p* < 0.01 (two-way ANOVA for GSK126 in neurons), *p* < 0.05 (two-way ANOVA for GSK343 in neurons); *n* = 3 per group. All data represent mean ± SEM.

Because our initial experiments were conducted using heterogeneous brain tissue, we next compared the expression levels of *Tet1^FL^* and *Tet1^S^* in postnatal hippocampal neurons and glia using qRT-PCR. *Tet1^S^* was expressed at ∼3-fold higher levels in neurons than in glia (*Tet1^S^* fold change: glia, 0.34 ± 0.017 vs neurons, 1 ± 0.14, *t*_(10)_ = 5, *p* = 0.0005, *n* = 6 all groups; unpaired two-tailed *t* test), whereas *Tet1^FL^* transcripts were ∼15-fold more abundant in glia than in neurons (*Tet1^FL^* fold change: glia, 15 ± 0.74 vs neurons, 1.2 ± 0.32, *t*_(10)_ = 17, *p* < 0.0001, *n* = 6 all groups; unpaired two-tailed *t* test; Fig. 2*A*). To explore these differences further, we compared the chromatin status of each *Tet1* isoform promoter in non-neuronal (NeuN–) cells to that of neurons using previously published ChIP-seq datasets from adult hippocampal tissue (Halder et al., 2016). Similar to neurons, we found in NeuN– cells that both *Tet1* isoform promoters were co-enriched for H3K4me3, H3K9ac, and H3K27ac. However, in these cells, the most enriched H3K4me3 peak was located at the *Tet1^FL^* promoter (Fig. 2*B*), while in neurons we observed the strongest enrichment at the *Tet1^S^* promoter (Fig. 1*A*). In addition, we found that in neurons, the *Tet1^FL^* promoter was also marked by the repressive histone modification H3K27me3 (Fig. 2*C*). The presence of active (H3K4me3 and H3K9ac) and repressive (H3K27me3) histone marks at *Tet1^FL^* exon 1 in neurons suggests that the *Tet1^FL^* promoter is bivalent in these cells, which has been shown to keep genes expressed at low basal levels, poised for reactivation (Bernstein et al., 2006). To test whether H3K27me3-mediated silencing accounted for differences in *Tet1^FL^* expression between glia and neurons, we blocked formation of the repressive mark using the two histone-lysine N-methyltransferase Enhancer of Zeste Homolog 2 (EZH2) inhibitors, GSK126 and GSK343 (Verma et al., 2012; Huang et al., 2019). As predicted by our chromatin analysis, both drugs significantly increased *Tet1^FL^* transcript levels in neurons (GSK126: *F*_(2,12)_ = 8, *p* = 0.0062; two-way ANOVA; *Tet1^FL^*: DMSO, 1 ± 0.15 vs 1.25 μm, 1.8 ± 0.18, *p* = 0.011, and DMSO, 1 ± 0.15 vs 2.5 μm, 2.6 ± 0.31, *p* < 0.001; Dunnett's *post hoc* test; GSK343: *F*_(2,12)_ = 4.3, *p* = 0.039; two-way ANOVA; *Tet1^FL^*: DMSO, 1 ± 0.13 vs 1.25 μm, 2.8 ± 0.58, *p* = 0.0062, and DMSO, 1 ± 0.13 vs 2.5 μm, 3 ± 0.6, *p* = 0.0032; *n* = 3 all groups; Dunnett's *post hoc* test; Fig. 2*D*,*E*), while in glia its expression remained unchanged (GSK126: *F*_(2,12)_ = 0.69, *p* = 0.52: GSK343: *F*_(2,12)_ = 0.91, *p* = 0.43; two-way ANOVA; Fig. 2*F*,*G*). Expression of *Tet1^S^* was unaffected by the EZH2 inhibitors in either cell type (GSK126, neurons: DMSO, 1 ± 0.10 vs 1.25 μm, 0.98 ± 0.07, *p* = 0.99, and DMSO, 1 ± 0.10 vs 2.5 μm, 0.71 ± 0.03, *p* = 0.4, *n* = 3 all groups; Dunnett's *post hoc* test; glia: DMSO, 1 ± 0.03 vs 1.25 μm, 1.2 ± 0.0091, *p* = 0.15, and DMSO, 1 ± 0.030 vs 2.5 μm, 1.1 ± 0.047, *p* = 0.51, *n* = 3 all groups; Dunnett's *post hoc* test: GSK343, neurons: DMSO, 1 ± 0.07 vs 1.25 μm, 0.90 ± 0.093, *p* = 0.97, and DMSO, 1 ± 0.07 vs 2.5 μm, 0.85 ± 0.07, *p* = 0.94, *n* = 3 all groups; Dunnett's *post hoc* test; glia: DMSO, 1 ± 0.08 vs 1.25 μm, 1 ± 0.052, *p* = 0.96, and DMSO, 1 ± 0.08 vs 2.5 μm, 1.1 ± 0.02, *p* = 0.46, *n* = 3 all groups; Dunnett's *post hoc* test; Fig. 2*D*–*G*). Together, these results demonstrate that *Tet1* isoform transcript usage differs significantly between neurons and glia, and that *Tet1^FL^* expression is specifically suppressed in neurons through epigenetic mechanisms involving EZH2-generated H3K27me3 marks.

### *Tet1^S^* transcript levels are downregulated in response to neuronal activity

**Figure 3.**
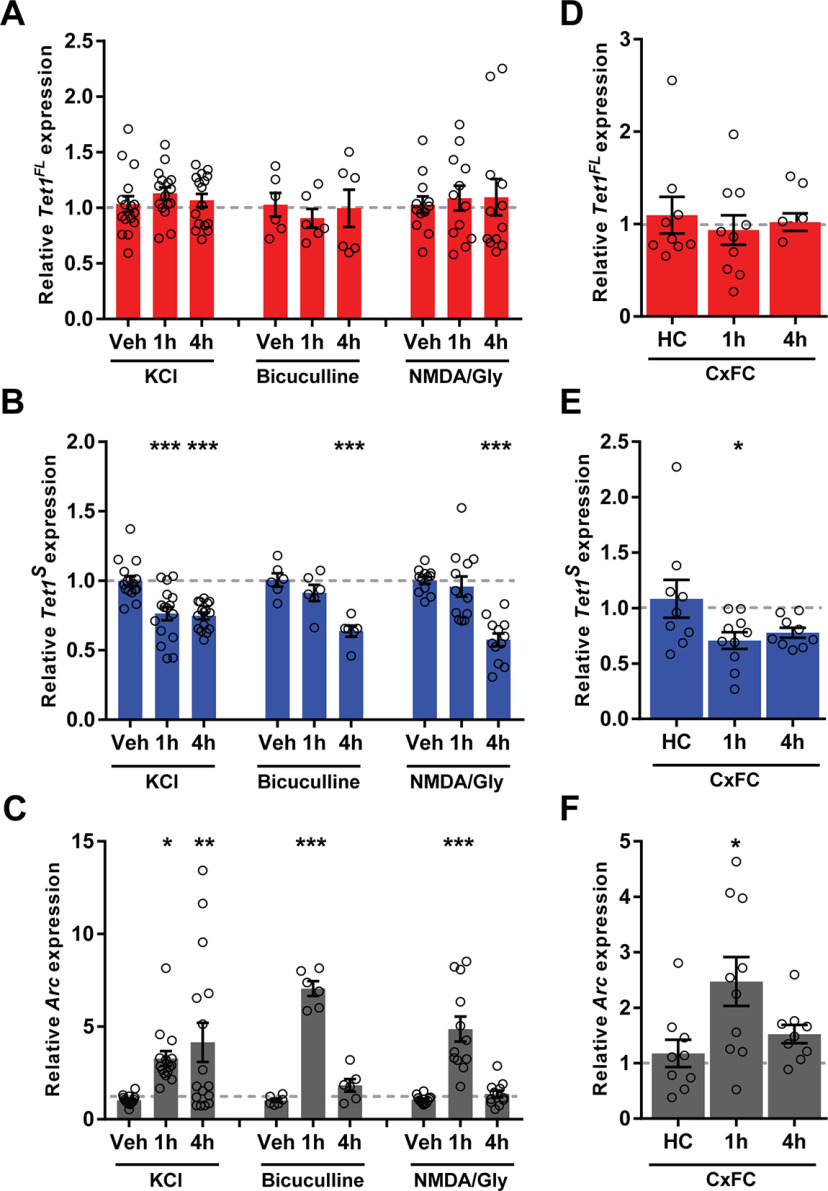
Neuronal activity-dependent downregulation of *Tet1^S^* transcript levels. qRT-PCR analysis of (***A***) *Tet1^FL^*, (***B***) *Tet1^S^*, and (***C***) *Arc* expression levels in primary hippocampal neurons 1 and 4 h after stimulation with 25 mm KCl, 50 μm bicuculline, or a combination of 10 μm NMDA + 2 μm glycine; *p < 0.05, **p < 0.01, ***p < 0.001 (Dunnett's *post hoc* test vs vehicle); *p* < 0.001 (one-way ANOVA). KCl, *n* = 15–16 per group; bicuculline, *n* = 6 per group; NMDA and glycine, *n* = 12 per group. qRT-PCR analysis of (***D***) *Tet1^FL^*, (***E***) *Tet1^S^*, and (***F***) *Arc* expression levels in area hippocampal CA1 1 and 4 h after CxFC (three context-shock pairings, 0.75 mA, 2 s); *p < 0.05 (Dunnett's *post hoc* test vs home cage); *p* < 0.05 (one-way ANOVA); *n* = 9–10 mice per group. All data represent mean ± SEM.

We and others previously reported that total *Tet1* mRNA levels are decreased in response to neuronal activity (Kaas et al., 2013; Widagdo et al., 2014). To examine the contributions of the *Tet1^FL^* and *Tet1^S^* transcripts to these changes, hippocampal neuron cultures were incubated for 1 or 4 h with KCl, bicuculline, or NMDA/glycine, and expression levels were evaluated using qRT-PCR. We found that *Tet1^FL^* mRNA levels were unaffected by any of these treatments (*Tet1^FL^* fold change, KCl: *F*_(2,45)_ = 0.6, *p* = 0.55; bic: *F*_(2,15)_ = 0.25, *p* = 0.78; NMDA/gly: *F*_(2,33)_ = 0.091; one-way ANOVA; Fig. 3*A*). In contrast, *Tet1^S^* mRNA levels were significantly decreased at both the 1- and 4-h time points after KCl stimulation (*Tet1^S^* fold change: *F*_(2,45)_ = 15, *p* < 0.0001; one-way ANOVA; Veh, 1 ± 0.034, *n* = 16 vs 1 h, 0.76 ± 0.048, *n* = 15, *p* < 0.0001, and Veh, 1 ± 0.034, *n* = 16 vs 4 h, 0.75 ± 0.024, *n* = 16, *p* < 0.0001; Dunnett's *post hoc* test) and at the 4-h time point after treatment with either bicuculline (*Tet1^S^* fold change: *F*_(2,15)_ = 15, *p* = 0.0003; one-way ANOVA; Veh, 1 ± 0.048, *n* = 6 vs 4 h, 0.64 ± 0.041, *n* = 6, *p* = 0.0002; Dunnett's *post hoc* test) or NMDA/gly (fold change: *F*_(2,33)_ = 21, *p* < 0.0001; one-way ANOVA; Veh, 1 ± 0.025, *n* = 12 vs 4 h, 0.58 ± 0.045, *n* = 12, *p* < 0.0001; Dunnett's *post hoc* test), suggesting that its expression is regulated by activity-dependent and NMDA receptor-dependent mechanisms (Fig. 3*B*). We confirmed that each of these treatments significantly increased expression levels of the immediate early gene (IEG) *activity regulated cytoskeleton associated protein* (*Arc*), as expected (*Arc* fold change, KCl: *F*_(2,44)_ = 6.034, *p* = 0.0048; one-way ANOVA; Veh, 1 ± 0.063, *n* = 16 vs 1 h, 3.3 ± 0.4, *n* = 15, *p* = 0.04, and Veh, 1 ± 0.063, *n* = 16 vs 4 h, 4.2 ± 1.1, *n* = 16, *p* = 0.0030; Dunnett's *post hoc* test: bic: *F*_(2,15)_ = 113.4, *p* < 0.0001; one-way ANOVA; Veh, 1 ± 0.097, *n* = 6 vs 1 h, 7.1 ± 0.4, *n* = 6, *p* < 0.0001; Dunnett's *post hoc* test: NMDA/gly: *F*_(2,33)_ = 27.02, *p* < 0.0001; one-way ANOVA; Veh, 1.1 ± 0.064, *n* = 12 vs 1 h, 4.9 ± 0.68, *n* = 12, *p* < 0.0001; Dunnett's *post hoc* test; Fig. 3*C*).

Next, we measured *Tet1^FL^* and *Tet1^S^* expression levels in tissue extracted from hippocampal area CA1 after CxFC to examine whether both isoforms responded similarly to neuronal activity induced *in vivo* during memory formation. Again, we observed that *Tet1^FL^* transcript levels remained unchanged (*Tet1^FL^* fold change: *F*_(2,25)_ = 0.2666, *p* = 0.77; one-way ANOVA; Fig. 3*D*), whereas *Tet1^S^* expression was significantly downregulated 1 h after training (*Tet1^S^* fold change: *F*_(2,25)_ = 3.399, *p* = 0.0494; one-way ANOVA; hc, 1.1 ± 0.17, *n* = 9 vs 1 h, 0.71 ± 0.075, *n* = 10, *p* = 0.037; Dunnett's *post hoc* test; Fig. 3*E*). As in primary cultures, *Arc* expression was significantly induced in CA1 after CxFC (*Arc* fold change: *F*_(2,25)_ = 4.55, *p* = 0.021, one-way ANOVA; hc, 1.2 ± 0.25, *n* = 9 vs 1 h, 2.5 ± 0.44, *n* = 10, *p* = 0.015; Dunnett's *post hoc* test; Fig. 3*F*). Together, these results show that transcript levels of the *Tet1^S^* gene are generally downregulated in response to hippocampal neuron stimulation, suggesting that under basal conditions the isoform may act as a molecular restraint on activity-dependent processes in neurons.

### Individual manipulation of *Tet1^FL^* and *Tet1^S^* expression in hippocampal neurons

**Figure 4.**
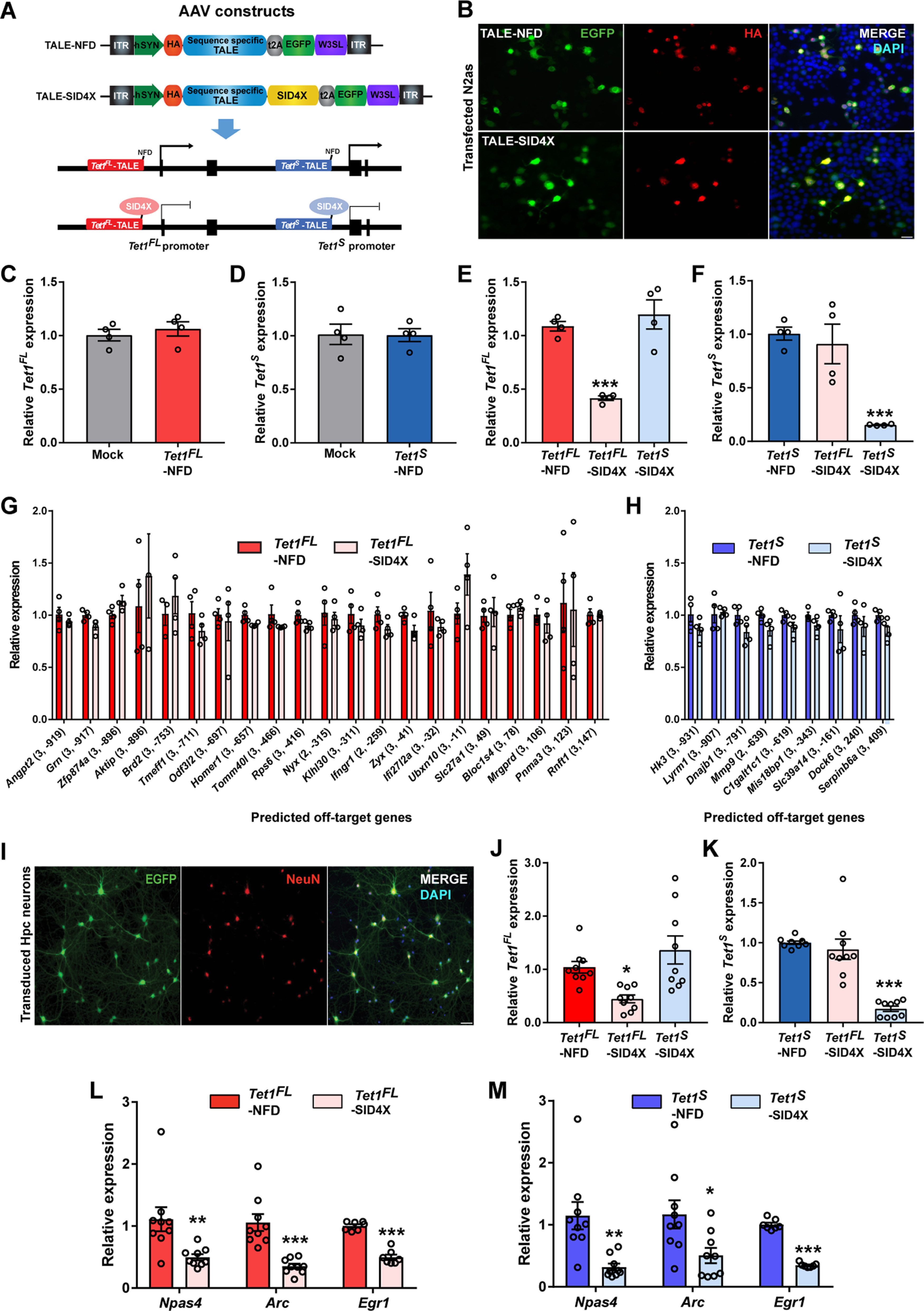
Construction and validation of synthetic transcription factors to repress endogenous *Tet1* isoform expression in neurons. ***A***, top, Illustration of neuron-specific TALE-construct modifications to increase target sequence capacity. ITR, inverted terminal repeats; hSYN, human synapsin 1 promoter; HA, human influenza hemagglutinin tag; TALE, transcriptional activator like effector; t2A, thosea asigna virus 2A (self-cleaving peptide); SID4X, 4 copies of the mSin3 interacting domain; EGFP, enhanced green fluorescent protein; W3SL, truncated woodchuck hepatitis posttranscriptional regulatory element and polyadenylation signal cassette (Choi et al., 2014). Bottom, Strategy to transcriptionally repress *Tet1^FL^* or *Tet1^S^* using TALE-SID4X constructs. ***B***, Representative images of GFP and HA immunostaining in N2a cells expressing TALE-NFD, or -SID4X constructs. Scale bar: 20 μm. ***C***, qRT-PCR analysis of *Tet1^FL^* expression levels in mock and *Tet1^FL^*-NFD transfected N2a cells; *n* = 4 per group. ***D***, qRT-PCR analysis of *Tet1^S^* expression levels in mock and *Tet1^S^-*NFD-transfected N2a cells; *n* = 4 per group. ***E***, qRT-PCR analysis of *Tet1^FL^* expression levels in N2a cells transfected with *Tet1^FL^*-NFD, *Tet1^FL^*-SID4X or *Tet1^S^*-SID4X; ***p < 0.001 (Dunnett's *post hoc* test); *p* < 0.05 (one-way ANOVA); *n* = 4. ***F***, qRT-PCR analysis of *Tet1^S^* expression levels in N2a cells transfected with *Tet1^S^*-NFD, *Tet1^FL^*-SID4X, or *Tet1^S^*-SID4X; ***p < 0.001 (Dunnett's *post hoc* test); *p* < 0.05 (one-way ANOVA); *n* = 4. ***G***, qRT-PCR analysis of predicted off-target genes for the *Tet1^FL^* TALE target sequence. (*x*-axis key: # mismatches, distance relative to TSS); *n* = 3–4 per group. Statistical outcomes in Extended Data Figure 4-1. ***H***, qRT-PCR analysis of predicted off-target genes for the *Tet1^S^* TALE target sequence (*x*-axis key: # mismatches, distance relative to TSS); *n* = 3–4 per group. Statistical outcomes in Extended Data Figure 4-2. ***I***, Representative EGFP and NeuN immunostaining of primary hippocampal cultures 5 d after transduction with AAV1-TALE constructs. Scale bar: 50 μm. ***J***, qRT-PCR analysis of *Tet1^FL^* expression levels in primary hippocampal neurons transduced with AAV1-*Tet1^FL^*-NFD, -*Tet1^FL^*-SID4X, or -*Tet1^S^*-SID4X; *p < 0.05 (Dunnett's *post hoc* test); *p* < 0.05 (one-way ANOVA); *n* = 9 per group. ***K***, qRT-PCR analysis of *Tet1^S^* expression levels in primary hippocampal neurons transduced with AAV1-*Tet1^S^*-NFD, -*Tet1^FL^*-SID4X, or -*Tet1^S^*-SID4X; ***p < 0.001 (Dunnett's *post hoc* test); *p* < 0.05 (one-way ANOVA); *n* = 9 per group. ***L***, qRT-PCR analysis of *Npas4*, *Arc*, and *Egr1* expression levels in primary hippocampal neurons transduced with AAV1-*Tet1^FL^*-NFD and -*Tet1^FL^*-SID4X; **p < 0.01, ***p < 0.001 (unpaired two-tailed *t* test); *n* = 9 per group. ***M***, qRT-PCR analysis of *Npas4*, *Arc*, and *Egr1* expression levels in primary hippocampal neurons transduced with AAV1-*Tet1^S^*-NFD and -*Tet1^S^*-SID4X; *p < 0.05, **p < 0.01, ***p < 0.001 (unpaired two-tailed *t* test); *n* = 9 per group. All data represent mean ± SEM.

The differential regulation of *Tet1* isoform expression following neuronal activation suggests that *Tet1^FL^* and *Tet1^S^* may have unique cell-specific functions. However, prior studies globally manipulated the expression of both genes, providing limited insight into the cell-specific functions of each *Tet1* isoform. Therefore, we developed genetic tools to selectively manipulate *Tet1^FL^* and *Tet1^S^* expression levels in neurons, both in culture and *in vivo*. To accomplish this, we designed sequence-programmable TALEs to selectively target *Tet1^FL^* or *Tet1^S^* because of their previously reported high target specificity, cell type-specific expression, and small size compatible with *in vivo* delivery using a single AAV virus (Konermann et al., 2013; Mendenhall et al., 2013; Juillerat et al., 2014; Polstein et al., 2015). HA-tagged TALEs were designed to specifically bind to DNA sequences at each *Tet1* isoform promoter and either repress transcription (TALE-SID4X, four copies of the mSin3 interacting domain) or serve as a target sequence-specific control (TALE-NFD, no functional domain; Konermann et al., 2013; Choi et al., 2014; Fig. 4*A*,*B*). TALE expression in these modified AAV vectors was placed under control of the human synapsin I promoter (hSYN), which drives expression only in neurons (Kügler et al., 2003). We found that expression of either control TALE in N2a cells, hereafter referred to as *Tet1^FL^*-NFD and *Tet1^S^*-NFD, did not alter the expression of the *Tet1* isoforms relative to mock-transfected cells, suggesting that TALE binding to these promoter regions without an effector domain did not sterically hinder transcription (fold changes: mock, 1 ± 0.095 vs *Tet1^FL^*-NFD, 1 ± 0.061, *t*_(6)_ = 0.069, *p* = 0.95, and mock, 1 ± 0.053 vs *Tet1^S^*-NFD, 1.1 ± 0.067, *t*_(6)_ = 0.68, *p* = 0.52, *n* = 4 all groups; unpaired two-tailed *t* tests; Fig. 4*C*,*D*). Conversely, expression of the TALE repressors, hereafter referred to as *Tet1^FL^*-SID4X and *Tet1^S^*-SID4X, significantly inhibited the expression of *Tet1^FL^* and *Tet1^S^* in N2a cells, respectively. Importantly, neither TALE repressor affected transcript levels of the opposite isoform, indicating isoform-specific targeting (*Tet1^FL^* fold changes: *F*_(2,9)_ = 26, *p* = 0.0002; one-way ANOVA; *Tet1^FL^*-NFD, 1.1 ± 0.044 vs *Tet1^FL^*-SID4X, 0.42 ± 0.021, *p* = 0.0006, and *Tet1^FL^*-NFD, 1.1 ± 0.044 vs *Tet1^S^*-SID4X, 1.2 ± 0.14, *p* = 0.57, *n* = 4 all groups; Dunnett's *post hoc* test: *Tet1^S^* fold changes: *F*_(2,9)_ = 17, *p* = 0.0008; one-way ANOVA; *Tet1^S^*-NFD, 1 ± 0.061 vs *Tet1^S^*-SID4X, 0.15 ± 0.0039, *p* = 0.0009, and *Tet1^S^*-NFD, 1 ± 0.061 vs *Tet1^FL^*-SID4X, 0.91 ± 0.18, *p* = 0.78, *n* = 4 all groups; Dunnett's *post hoc* test; Fig. 4*E*,*F*). In addition, we measured the effects of *Tet1^FL^*-SID4X and *Tet1^S^*-SID4X on the expression of any predicted off-target genes that contained either TALE sequence up to three mismatches within +1000 to −400 bases of its TSS. Genes identified as TET1 targets in the ChIP enrichment analysis (ChEA) transcription factor targets dataset were excluded from the analysis (Lachmann et al., 2010). We found that expression of either TALE repressor did not result in any significant changes in the transcript levels of their respective off-target genes, providing further evidence of their specificity (Fig. 4*G*,*H*; Extended Data Figs. 4-1, 4-2). Next, to examine the function of the *Tet1* isoform-specific TALEs in primary cells, we packaged each construct into AAV1 viral particles and transduced hippocampal neurons to investigate their efficacy and specificity. Immunocytochemistry revealed that expression of the TALE constructs was neuron specific, as only cells positive for the neuronal marker NeuN expressed EGFP (Fig. 4*I*). Similar to their effects in N2a cells, transduction of primary hippocampal neurons with AAV1-*Tet1^FL^*-SID4X and *Tet1^S^*-SID4X led to a significant reduction in the expression levels of their intended *Tet1* isoform target without affecting the opposite transcript (*Tet1^FL^* fold changes: *F*_(2,24)_ = 7.549, *p* = 0.0029; one-way ANOVA; *Tet1^FL^*-NFD, 1 ± 0.11 vs *Tet1^FL^*-SID4X, 0.45 ± 0.073, *p* = 0.039, and *Tet1^FL^*-NFD, 1 ± 0.11 vs *Tet1^S^*-SID4X, 1.4 ± 0.26, *p* = 0.31, *n* = 9 all groups; Dunnett's *post hoc* test: *Tet1^S^* fold changes: *F*_(2,24)_ = 35, *p* < 0.0001; one-way ANOVA; *Tet1^S^*-NFD, 1 ± 0.02 vs *Tet1^S^*-SID4X, 0.17 ± 0.033, *p* < 0.0001, and *Tet1^S^*-NFD, 1 ± 0.02 vs *Tet1^FL^*-SID4X, 0.92 ± 0.13, *p* = 0.66, *n* = 9 all groups; Dunnett's *post hoc* test; Fig. 4*J*,*K*). Finally, we measured transcript levels of the IEGs *Npas4*, *Arc*, and *Egr1* in primary hippocampal neurons transduced with AAV1-*Tet1^FL^*-SID4X or *Tet1^S^*-SID4X because TET1 was previously shown to regulate their expression in the brain (Kaas et al., 2013; Rudenko et al., 2013; Kumar et al., 2015; Towers et al., 2018). We found that SID4X-mediated repression of either *Tet1* isoform led to a significant reduction in the expression of all three genes compared with NFD controls (*Tet1^FL^*-NFD vs *Tet1^FL^*-SID4X fold changes: *Npas4*, 1.1 ± 0.19 vs 0.38 ± 0.041, *t*_(16)_ = 3.7, *n* = 9, *p* = 0.002; *Arc*, 1.1 ± 0.14, vs 0.35 ± 0.04, *t*_(16)_ = 5, *n* = 9, *p* < 0.0001; *Egr1*, 1 ± 0.027, vs 0.5 ± 0.043, *t*_(12)_ = 10, *n* = 7, *p* < 0.0001: *Tet1^S^*-NFD vs *Tet1^S^*-SID4X fold changes: *Npas4*, 1.1 ± 0.22 vs 0.32 ± 0.057 *t*_(16)_ = 3.6, *n* = 9, *p* = 0.0023; *Arc*, 1.2 ± 0.23 vs 0.51 ± 0.12, *t*_(16)_ = 2.6, *n* = 9, *p* = 0.021; *Egr1*, 1 ± 0.032 vs 0.35 ± 0.014, *t*_(12)_ = 19, *n* = 7, *p* < 0.0001; unpaired two tailed *t* tests; Fig. 4*L*,*M*). Together, these results demonstrate that our modified TALE tools significantly repress the transcription of each individual *Tet1* isoform, are neuron specific, and result in changes in the expression of genes previously shown to be targets of TET1 in the CNS.

10.1523/JNEUROSCI.1821-20.2020.f4-1 Extended Data Figure 4-1 Statistical analysis of expression changes in predicted *Tet1^FL^* TALE sequence off-target genes. Unpaired two-tailed student *t* tests related to Figures 4*G*. *Tet1^FL^* predicted off-target genes. AAV1-*Tet1****^FL^***-NFD versus -*Tet1****^FL^***-SID4X; *n* = 3–4 per group. Download Figure 4-1, XLSX file.

10.1523/JNEUROSCI.1821-20.2020.f4-2 Extended Data Figure 4-2 Statistical analysis of expression changes in predicted *Tet1^S^* TALE sequence off-target genes. Unpaired two-tailed student *t* tests related to Figure 4*H*. AAV1-*Tet1^S^*-NFD versus -*Tet1^S^*-SID4X; *n* = 3–4 per group. Download Figure 4-2, XLSX file.

### *Tet1^FL^* and *Tet1^S^* regulate unique subsets of the neuronal transcriptome

**Figure 5.**
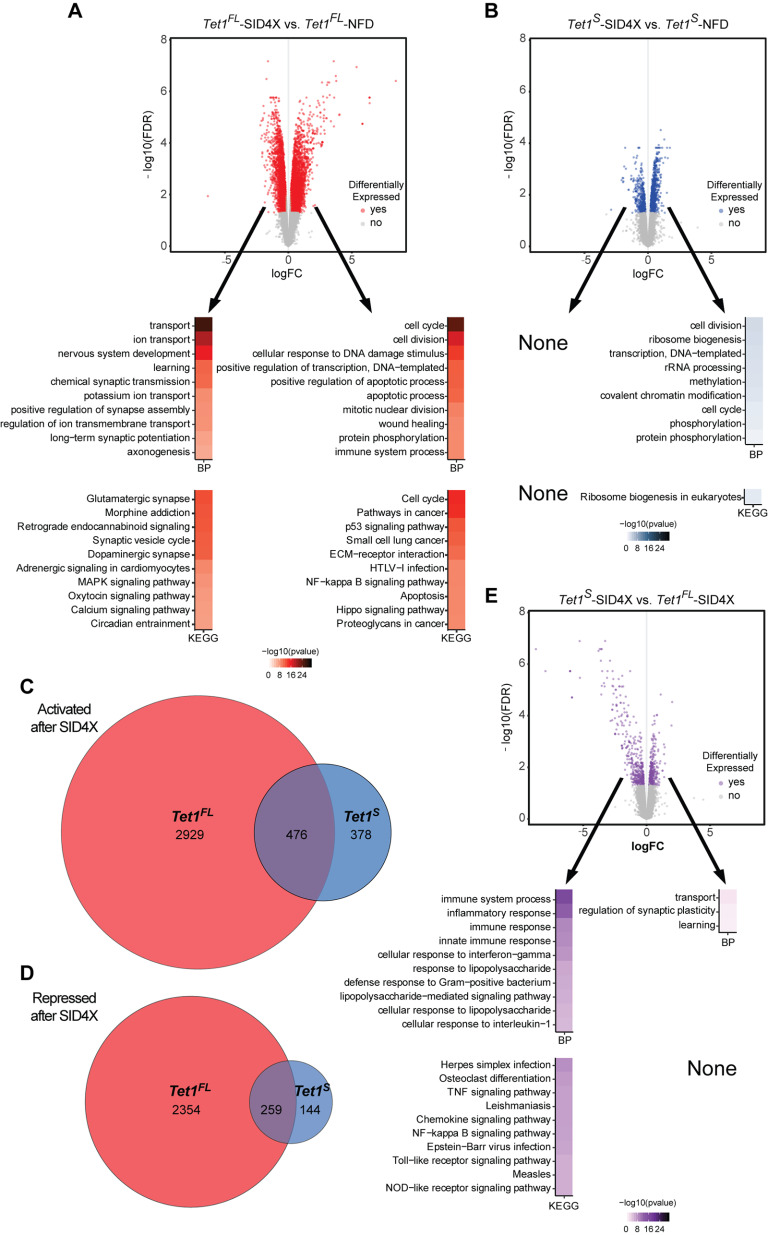
Transcriptomic analysis of hippocampal neuron cultures depleted of *Tet1^FL^* and *Tet1^S^*. ***A***, Top, Volcano plot analysis of genome wide RNA-seq data comparing AAV1-*Tet1^FL^*-NFD and *Tet1^FL^*-SID4X transduced hippocampal neurons (±0.2 log2FC, FDR < 0.05); *n* = 3 biological replicates. Extended Data Figure 5-1, all genes; Extended Data Figures 5-2, 5-3, downregulated and upregulated DEGs, respectively. Bottom, Ten most statistically significant BP and KEGG enrichment terms associated with upregulated and downregulated DEGs in *Tet1^FL^*-SID4X versus *Tet1^FL^*-NFD transduced hippocampal neurons; *p* < 0.05 (Benjamini–Hochberg corrections). Extended Data Figures 5-2, 5-3, all BP and KEGG terms associated with downregulated and upregulated DEGs, respectively. ***B***, Volcano plot analysis of genome wide RNA-seq data comparing AAV1-*Tet1^S^*-NFD and -*Tet1^S^*-SID4X transduced hippocampal neurons (±0.2 log2FC, FDR < 0.05); *n* = 3 biological replicates. Extended Data Figure 5-4, all genes; Extended Data Figures 5-5, 5-6, downregulated and upregulated DEGs, respectively. Bottom, Statistically significant BP and KEGG enrichment terms of upregulated DEGs in AAV1-*Tet1^S^*-SID4X versus -*Tet1^S^*-NFD transduced hippocampal neurons. None, no statistically significant GO terms identified; *p* < 0.05 (Benjamini–Hochberg corrections). Extended Data Figures 5-5, 5-6, all BP and KEGG terms associated with downregulated and upregulated DEGs, respectively. ***C***, ***D***, Overlapping genes upregulated and downregulated, respectively, in AAV1-*Tet1^S^*-SID4X versus -*Tet1^FL^*-SID4X transduced hippocampal neurons. Extended Data Figure 5-7, lists of overlapping downregulated and upregulated DEGs. ***E***, top, Volcano plot analysis of genome wide RNA-seq data comparing AAV1-*Tet1^S^*-SID4X and -*Tet1^FL^*-SID4X transduced hippocampal neurons (±0.2 log2FC, FDR < 0.05); *n* = 3 biological replicates. Extended Data Figure 5-8, all genes; Extended Data Figures 5-9, 5-10, downregulated and upregulated DEGs, respectively. Bottom, Ten most statistically significant BP and KEGG enrichment terms associated with upregulated and downregulated DEGs in AAV1-*Tet1^S^*-SID4X vs -*Tet1^FL^*-SID4X transduced hippocampal neurons; *p* < 0.05 (Benjamini–Hochberg corrections). Extended Data Figures 5-9, 5-10, all BP and KEGG terms associated with downregulated and upregulated DEGs, respectively.

To investigate the effects of each individual *Tet1* isoform on neuronal gene expression, we infected postnatal hippocampal neurons with our AAV1-*Tet1^FL^*-SID4X, -*Tet1^FL^-*NFD, -*Tet1^S^*-SID4X, or -*Tet1^S^*-NFD and performed an unbiased, transcriptome-wide RNA-seq analysis on control and *Tet1* isoform-depleted cells. We found that despite its low transcript levels, acute repression of *Tet1^FL^* caused widespread transcriptional changes in neurons. Using a cutoff greater than ±0.2 log2 fold-change (log2FC) and an FDR < 0.05, we identified >6000 differentially expressed genes (DEGs; Fig. 5*A*, top; Extended Data Fig. 5-1). Gene Ontology (GO) analysis revealed that *Tet1^FL^-*modulated mRNAs functioned in a wide assortment of biological processes (BP) and Kyoto Encyclopedia of Genes and Genomes pathways (KEGG; Fig. 5*A*, bottom; Extended Data Figs. 5-2, 5-3). In both sets of analyses, genes downregulated in response to *Tet1^FL^* repression were generally enriched for neuron-associated functions, such as ion transport, learning, long-term synaptic potentiation and the synaptic vesicle cycle (BP terms and KEGG terms: ion transport: 3.3 × 10^−20^, learning: 3.6 × 10^−12^, long-term synaptic potentiation: 7.8 × 10^−8^, synaptic vesicle cycle: 1.2 × 10^−12^; Benjamini–Hochberg adjusted *p* values; Fig. 5*A*, bottom left). In contrast, upregulated genes were enriched for BP terms associated with the cell cycle, DNA damage, and immune function (6.3 × 10^−25^, 1.6 × 10^−14^, 1.3 × 10^−9^, respectively; Benjamini–Hochberg adjusted *p* values) as well as several KEGG pathway categories related to cancer pathways, extracellular matrix (ECM) receptor interactions, and NF-κB signaling (3.5 × 10^−15^, 1.1 × 10^−11^, 7.3 × 10^−10^, respectively; Benjamini–Hochberg adjusted *p* values; Fig. 5*A*, bottom right). In the case of *Tet1^S^*, we found that its repression led to less than a quarter of the number of DEGs relative to *Tet1^FL^* (Fig. 5*B*, top; Extended Data Fig. 5-4). Genes upregulated following *Tet1^S^* repression were enriched for some of the same BP categories as *Tet1^FL^*, but also included terms associated with ribosomal biogenesis, methylation, and covalent chromatin modifications (5.1 × 10^−4^, 2 × 10^−3^, 3.7 × 10^−3^, respectively; Benjamini–Hochberg adjusted *p* values; Fig. 5*B*, bottom right; Extended Data Fig. 5-5). Notably, downregulated genes associated with *Tet1^S^* were not significantly enriched in any pathway or GO category (Extended Data Fig. 5-6). To further explore differences between *Tet1^FL^* and *Tet1^S^*-mediated transcriptional regulation, we performed a direct comparison of *Tet1^S^-*SID4X to *Tet1^FL^-*SID4X associated DEGs. Overall, we found that differentially expressed genes altered in response to the loss of each isoform did not entirely overlap, suggesting the two *Tet1* isoforms do not serve redundant functions in neurons. Slightly over half of activated genes (Fig. 5*C*; Extended Data Fig. 5-7) and repressed genes (Fig. 5*D*; Extended Data Fig. 5-7) after modulation of the short isoform were also changed after suppression of the long isoform. This prompted us to do a direct comparison of the SID4X conditions, and this further statistical analysis (Extended Data Fig. 5-8) revealed that DEGs expressed at lower levels in the *Tet1^S^*-SID4X dataset, relative to *Tet1^FL^*-SID4X, were generally involved in immune system regulation (BP terms; Immune system process: 1.7 × 10^−17^, inflammatory response: 2.2 × 10^−13^, innate immune response: 1.6 × 10^−9^: KEGG terms; TNF signaling pathway: 4.9 × 10^−8^, Nf-κB signaling pathway: 6.5 × 10^−8^; Benjamini–Hochberg adjusted *p* values; Fig. 5*E*, bottom left; Extended Data Fig. 5-9). Genes more abundantly expressed when *Tet1^S^* was repressed relative to *Tet1^FL^*, functioned in transport, regulation of synaptic plasticity, and learning (1.2 × 10^−2^, 3.3 × 10^−2^, 3.7 × 10^−2^, respectively; Benjamini–Hochberg adjusted *p* values; Fig. 5*E*, bottom right; Extended Data Fig. 5-10). We interpret this to mean that while overlaps exist, the acute repression of *Tet1^FL^* aberrantly activates inflammatory response pathways, while *Tet1^S^* does not. Moreover, relative to *Tet1^FL^*, the acute suppression of *Tet1^S^* elicits higher expression of genes involved in synaptic plasticity.

10.1523/JNEUROSCI.1821-20.2020.f5-1 Extended Data Figure 5-1 Expression of all genes in AAV1-*Tet1^FL^*-SID4X relative to -*Tet1^FL^*-NFD. Expression of all genes in a comparison of AAV1-*Tet1^FL^*-SID4X to -*Tet1^FL^*-NFD. Column headings: MSTRG, Stringtie ID or Refseq ID of gene (if gene prediction matches annotation exactly); logFC = log2 fold change; logCPM = average log2 counts per million; F = F statistic; PValue = probability value before multiple hypothesis correction; Refseq = best matching Refseq gene ID that best matches the stringtie predicted transcript; OGS = official gene symbol with period after to prevent date conversion in excel; Sig = whether the transcript passes the FDR and fold change cutoffs for significance. Download Figure 5-1, XLSX file.

10.1523/JNEUROSCI.1821-20.2020.f5-2 Extended Data Figure 5-2 DEGs significantly downregulated by AAV1-*Tet1^FL^*-SID4X relative to -*Tet1^FL^*-NFD. The subset of genes that were downregulated by repression of *Tet1^FL^*. To the right, the significantly enriched (Benjamini–Hochberg adjusted *p*s < 0.05) DAVID GO-enriched terms for BP (top) and KEGG pathways (bottom). Top 10 terms included in the main figure are bolded. Column headings: category, GO set utilized; Term, GO term title; Count, number of genes in gene set present in the genes that comprise the ontology term; %, percent of genes in the list of genes for a GO term; *p* value, probability value; Bonferroni, Bonferroni-corrected *p* value; Benjamini, Benjamini-corrected *p* value. Download Figure 5-2, XLSX file.

10.1523/JNEUROSCI.1821-20.2020.f5-3 Extended Data Figure 5-3 DEGs significantly upregulated by AAV1-*Tet1^FL^*-SID4X relative to -*Tet1^FL^*-NFD. The subset of genes that are upregulated by repression of *Tet1^FL^*. Download Figure 5-3, XLSX file.

10.1523/JNEUROSCI.1821-20.2020.f5-4 Extended Data Figure 5-4 Expression of all genes in AAV1-*Tet1^S^*-SID4X relative to -*Tet1^S^*-NFD. Expression of all genes in a comparison of AAV1-*Tet1^S^*-SID4X to -*Tet1^S^*-NFD. Download Figure 5-4, XLSX file.

10.1523/JNEUROSCI.1821-20.2020.f5-5 Extended Data Figure 5-5 DEGs significantly downregulated by AAV1-*Tet1^S^*-SID4X relative to -*Tet1^S^*-NFD. The subset of genes that are downregulated by repression of *Tet1^S^*. Download Figure 5-5, XLSX file.

**Figure 6.**
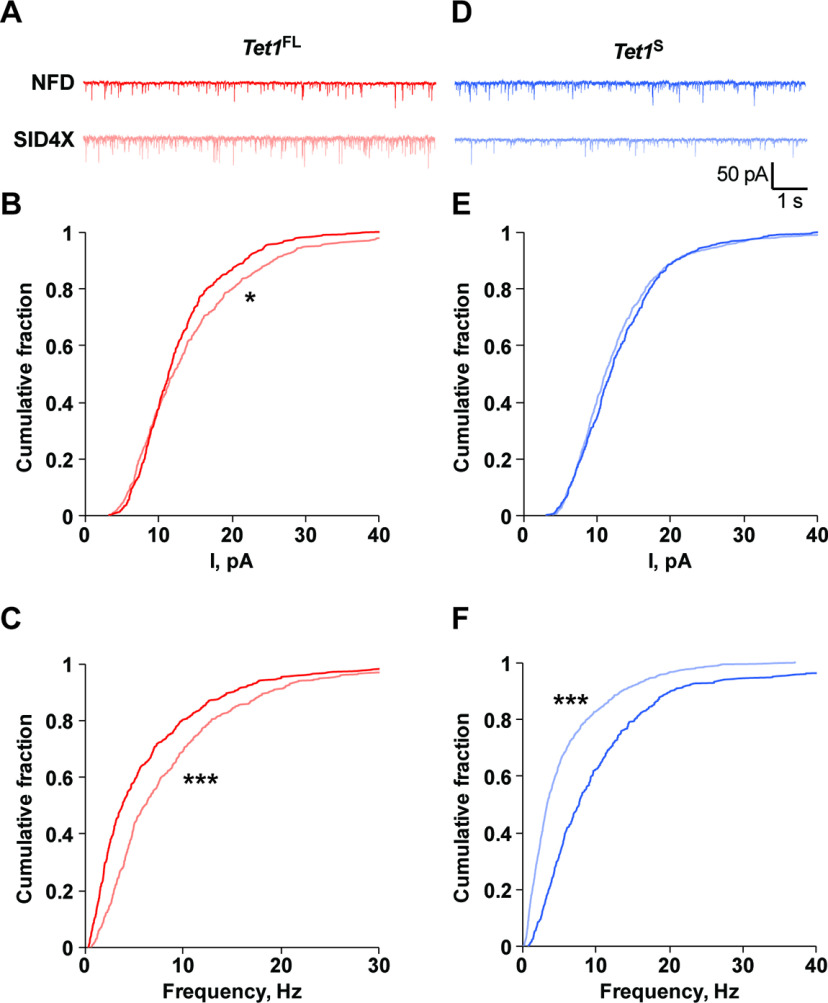
*Tet1^FL^* and *Tet1^S^* repression alters excitatory synaptic transmission. ***A***, Representative mEPSC traces from hippocampal neurons transduced with AAV1-*Tet1^FL^*-NFD and -*Tet1^FL^*-SID4X, calibration: 1 s, 50 pA. ***B***, Cumulative probability distribution plots of mEPSC amplitudes in hippocampal neurons transduced with AAV1-*Tet1^FL^*-NFD and -*Tet1^FL^*-SID4X; *p < 0.05 (Kolmogorov–Smirnov test); *n* = 19–20 cells per group. ***C***, Cumulative probability distribution plots of mEPSC frequencies in hippocampal neurons transduced with AAV1-*Tet1^FL^*-NFD and -*Tet1^FL^*-SID4X; ***p < 0.001 (Kolmogorov–Smirnov test); *n* = 19–20 cells per group. ***D***, Representative mEPSC traces from hippocampal neurons transduced with AAV1-*Tet1^S^*-NFD and -*Tet1^S^*-SID4X, calibration: 1 s, 50 pA. ***E***, Cumulative probability distribution plots of mEPSC amplitudes in hippocampal neurons transduced with AAV1-*Tet1^S^*-NFD and -*Tet1^S^*-SID4X; *p* > 0.05 (Kolmogorov–Smirnov test); *n* = 20–23 cells per group. ***F***, Cumulative probability distribution plots of mEPSC frequencies in hippocampal neurons transduced with AAV1-*Tet1^FL^*-NFD and -*Tet1^FL^*-SID4X; ***p < 0.001 (Kolmogorov–Smirnov test); *n* = 20–23 cells per group.

10.1523/JNEUROSCI.1821-20.2020.f5-6 Extended Data Figure 5-6 DEGs significantly upregulated by AAV1-*Tet1^S^*-SID4X relative to -*Tet1^S^*-NFD. The subset of genes that are upregulated by repression of *Tet1^S^*. Download Figure 5-6, XLSX file.

**Figure 7.**
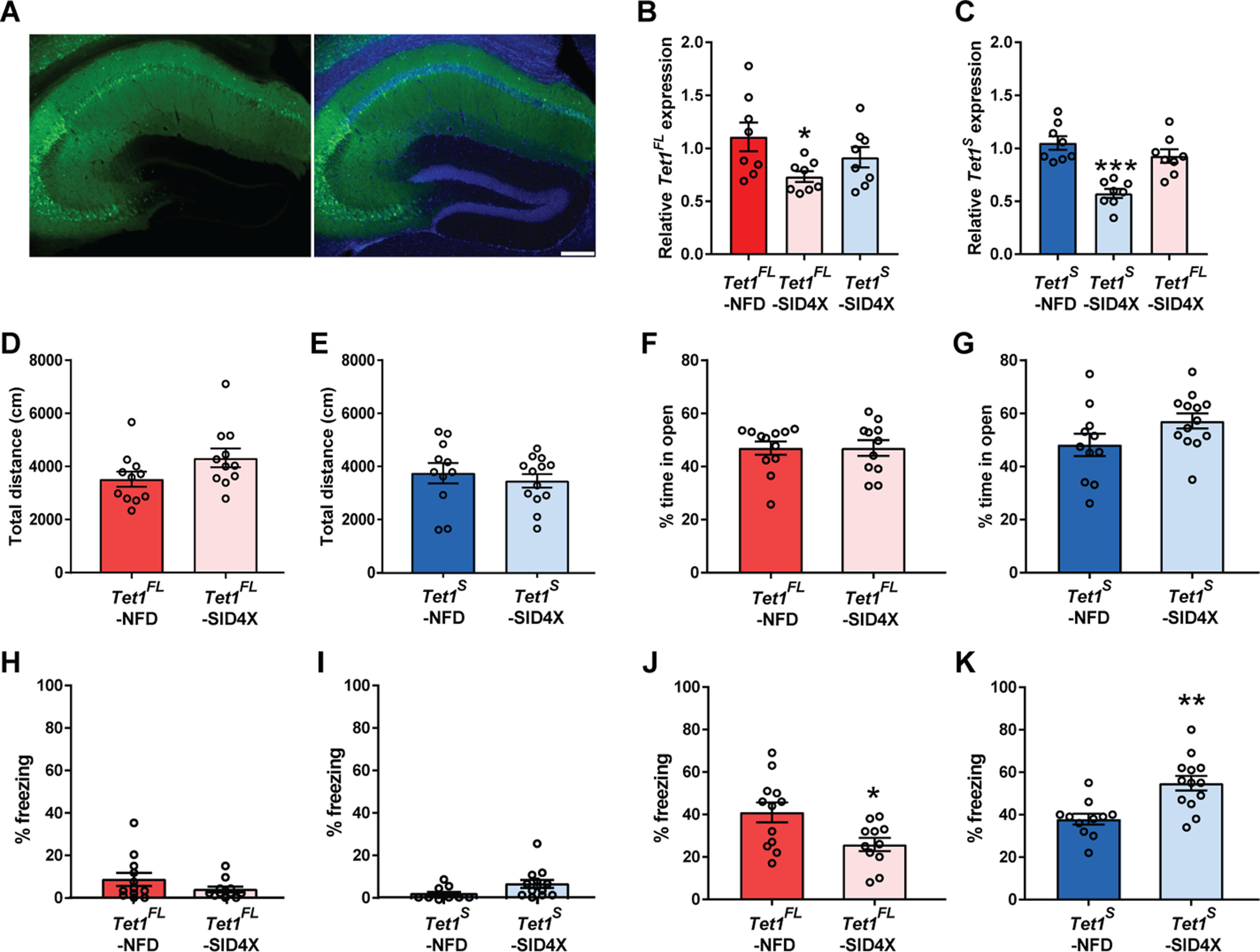
Neuron-specific *Tet1^FL^* and *Tet1^S^* repression differentially affect hippocampal-dependent memory formation. ***A***, left, Representative EGFP immunostaining in the dHPC two weeks after AAV1-mediated transduction of *Tet1* isoform-specific TALE constructs. Right, Merge of EGFP immunostaining and DAPI. Scale bar: 100 µm. ***B***, qRT-PCR analysis of *Tet1^FL^* expression levels in the dHPC two weeks after AAV1-*Tet1^FL^*-NFD, -*Tet1^FL^*-SID4X and -*Tet1^S^*-SID4X transduction; *p < 0.05 (Dunnett's *post hoc* test); *n* = 8. ***C***, qRT-PCR analysis of *Tet1^S^* expression levels in the dHPC two weeks after AAV1-*Tet1^S^*-NFD, -*Tet1^S^*-SID4X and -*Tet1^FL^*-SID4X transduction; ***p < 0.001 (Dunnett's *post hoc* test); *n* = 8 mice per group. ***D***, Total distance traveled (cm) during a 30-min open field exploration test in AAV1-*Tet1^FL^*-NFD and -*Tet1^FL^*-SID4X transduced mice; *n* = 11–12 mice per group. ***E***, Total distance traveled (cm) during a 30-min open field exploration test in AAV1-*Tet1^S^*-NFD and -*Tet1^S^*-SID4X transduced mice; *n* = 11–13 mice per group. ***F***, Percent time in the open arms during 5 min of exploration in the EZM in AAV1-*Tet1^FL^*-NFD and -*Tet1^FL^*-SID4X transduced mice; *n* = 11–12 mice per group. ***G***, Percent time in the open arms during 5 min of exploration in the EZM in AAV1-*Tet1^S^*-NFD and -*Tet1^S^*-SID4X transduced mice; *n* = 11–13 mice per group. ***H***, Percent time spent freezing in AAV1-*Tet1^FL^*-NFD and -*Tet1^FL^*-SID4X transduced mice during a 3.5-min CxFC training session; *n* = 11–12 mice per group. ***I***, Percent time spent freezing in AAV1-*Tet1^S^*-NFD and -*Tet1^S^*-SID4X transduced mice during a 3.5-min CxFC training session; *n* = 11–13 mice per group. ***J***, Percent time spent freezing in AAV1-*Tet1^FL^*-NFD and -*Tet1^FL^*-SID4X transduced mice during a 5-min CxFC test 24 h after training; *p < 0.05 (unpaired two-tailed *t* test); *n* = 11–12 mice per group. ***K***, Percent time spent freezing in AAV1-*Tet1^S^*-NFD and -*Tet1^S^*-SID4X transduced mice during a 5-min CxFC test 24 h after training; **p < 0.01 (unpaired two-tailed *t* test); *n* = 11–13 mice per group. All data represent mean ± SEM.

10.1523/JNEUROSCI.1821-20.2020.f5-7 Extended Data Figure 5-7 DEGs relative to each NFD control overlap. The genes that make up the total gene counts in the Venn diagrams in panels C and D. Download Figure 5-7, XLSX file.

10.1523/JNEUROSCI.1821-20.2020.f5-8 Extended Data Figure 5-8 Expression of all genes in AAV1-*Tet1^S^*-SID4X relative to -*Tet1^FL^*-SID4X. Expression of all genes in a direct comparison of AAV1-Tet1S-SID4X to AAV1-Tet1FL-SID4X. Download Figure 5-8, XLSX file.

10.1523/JNEUROSCI.1821-20.2020.f5-9 Extended Data Figure 5-9 DEGs significantly downregulated by AAV1-*Tet1^S^*-SID4X relative to -*Tet1^FL^*-SID4X. Significantly downregulated genes when repression of *Tet1^S^* is directly compared to repression of *Tet1^FL^*. Download Figure 5-9, XLSX file.

10.1523/JNEUROSCI.1821-20.2020.f5-10 Extended Data Figure 5-10 DEGs significantly upregulated by AAV1-*Tet1^S^*-SID4X relative to -*Tet1^FL^*-SID4X. Significantly upregulated genes when repression of *Tet1^S^* is directly compared to repression of *Tet1^FL^*. Download Figure 5-10, XLSX file.

### Acute repression of *Tet1^FL^* and *Tet1^S^* expression has opposing effects on synaptic transmission

Prior studies have found that *Tet1* KO animals do not exhibit altered hippocampal long-term potentiation (LTP; Rudenko et al., 2013; Kumar et al., 2015; Towers et al., 2018). However, shRNA-mediated KD of *Tet1* in neuron cultures has been shown to increase mEPSCs amplitudes, suggesting that the gene regulates at least some aspects of synaptic transmission (Yu et al., 2015). To examine whether *Tet1^FL^*, or *Tet1^S^*, might be responsible for these previously reported electrophysiological changes, we transduced hippocampal cultures with AAV1-TALEs using the same conditions as in Figure 4*J*,*K* and recorded mEPSCs in EGFP+ control and *Tet1* isoform-depleted neurons. We found that SID4X-mediated repression of *Tet1^FL^* significantly increased both mEPSC amplitude and frequency compared with controls (Fig. 6*A–C*), as reflected by significant rightward shifts in the cumulative probability distributions of both measurements (amplitude: *D* = 0.10, *p* = 0.012, frequency: *D* = 0.23, *p* < 0.0001, *Tet1^FL^*-NFD, *n* = 19, *Tet1^FL^*-SID4X, *n* = 20; Kolmogorov–Smirnov test). In contrast, *Tet1^S^* repression had no effect on mEPSC amplitude (Fig. 6*D*,*E*), but significantly reduced mEPSC frequency (Fig. 6*D*,*F*), illustrated by a significant leftward shift in the cumulative probability distribution (amplitude: *D* = 0.066, *p* = 0.15, frequency: *D* = 0.32, *p* < 0.0001, *Tet1^S^*-NFD, *n* = 20, *Tet1^S^*-SID4X, *n* = 23; Kolmogorov–Smirnov test). Overall, these data suggest that the acute and selective repression of *Tet1* isoform expression differentially regulates excitatory synaptic transmission.

### *Tet1^FL^* and *Tet1^S^* differentially regulate hippocampal-dependent memory

We next assessed the cognitive effects of selectively inhibiting *Tet1^FL^* and *Tet1^S^* expression in the dorsal hippocampus (dHPC) using our neuron-specific and isoform-specific molecular tools. Stereotaxic injection of *Tet1* isoform-specific AAV1-TALEs into the dHPC led to widespread Enhanced Green Fluorescent Protein (EGFP) expression throughout CA1-CA3 subfields after two weeks (Fig. 7*A*). Importantly, and similar to our findings *in vitro*, hippocampal transduction with AAV1-*Tet1^FL^*-SID4X and -*Tet1^S^*-SID4X lead to significant reductions in *Tet1^FL^* and *Tet1^S^*, respectively, without affecting the opposite isoform, suggesting that these molecular tools were also effective *in vivo* (*Tet1^FL^* fold changes: *F*_(2,21)_ = 3.49, *p* = 0.049; one-way ANOVA; *Tet1^FL^*-NFD, 1.1 ± 0.13 vs *Tet1^FL^*-SID4X, 0.73 ± 0.051, *p* = 0.028, and *Tet1^FL^*-NFD, 1.1 ± 0.13 vs *Tet1^S^*-SID4X, 0.91 ± 0.096, *p* = 0.31, *n* = 8 all groups; Dunnett's *post hoc* test: *Tet1^S^* fold changes: *F*_(2,21)_ = 18.45, *p* < 0.0001; one-way ANOVA; *Tet1^S^*-NFD, 1.05 ± 0.063 vs *Tet1^S^*-SID4X, 0.57 ± 0.044, *p* < 0.0001, and *Tet1^S^*-NFD, 1.05 ± 0.063 vs *Tet1^FL^*-SID4X, 0.92 ± 0.0.064, *p* = 0.25, *n* = 8 all groups; Dunnett's *post hoc* test; Fig. 7*B*,*C*).

We first tested whether transcriptional repression of *Tet1^FL^* and *Tet1^S^* in the dHPC led to any changes in locomotion or anxiety. In both the open field and Elevated Zero Maze (EZM) tests, there were no significant differences between mice infected with virus to silence either isoform compared with their isoform-specific controls [open field, total distance (cm): *Tet1^FL^*-NFD, 3520 ± 28, *n* = 12 vs *Tet1^FL^*-SID4X, 4322 ± 35, *n* = 11, *t*_(21)_ = 0.64, *p* = 0.53, and *Tet1^S^*-NFD, 3746 ± 38, *n* = 11 vs *Tet1^S^*-SID4X, 3459 ± 26, *n* = 13, *t*_(20)_ = 1.8, *p* = 0.0908; unpaired two-tailed *t* test: EZM, percent time in open: *Tet1^FL^*-NFD, 47 ± 2.5%, *n* = 12 vs *Tet1^FL^*-SID4X, 47 ± 3%, *n* = 11, *t*_(21)_ = 0.0041, *p* = 1, and *Tet1^S^*-NFD, 48 ± 4.2%, *n* = 11 vs *Tet1^S^*-SID4X, 57 ± 2.8%, *n* = 13, *t*_(22)_ = 1.8, *p* = 0.84; unpaired two-tailed *t* test], suggesting that manipulation of *Tet1^FL^* or *Tet1^S^* in the dHPC does not affect general locomotion (Fig. 7*D*,*E*) or basal anxiety levels (Fig. 7*F*,*G*).

Because previous studies examining the role of TET1 in memory formation were conducted in mice with disruptions that affect both isoforms (Rudenko et al., 2013; Zhang et al., 2013; Kumar et al., 2015; Towers et al., 2018), we next examined whether repression of either *Tet1^FL^* or *Tet1^S^* alone was sufficient to alter hippocampal-dependent memory formation. To test this, we trained mice using a moderate (see Materials and Methods) CxFC paradigm and tested them 24 h later, with the percentage of time spent freezing serving as an indirect measure of associative memory formation. We found no differences in the percentage of time freezing during training between *Tet1^FL^*-SID4X and *Tet1^FL^-*NFD mice (percent time freezing: *Tet1^FL^-*NFD, 8.1 ± 2.8%, *n* = 12 vs *Tet1^FL^*-SID4X, 3.6 ± 1.3%, *n* = 11, *t*_(21)_ = 1.4, *p* = 0.18; unpaired two-tailed *t* test), nor between the *Tet1^S^*-SID4X and *Tet1^S^*-NFD groups (percent time freezing: *Tet1^S^-*NFD, 3.6 ± 1.2%, *n* = 11 vs *Tet1^S^*-SID4X, 7 ± 1.8%, *n* = 13, *t*_(22)_ = 1.5, *p* = 0.14; unpaired two-tailed *t* test), suggesting depletion of *Tet1^FL^* or *Tet1^S^* in the dHPC does not affect baseline freezing levels (Fig. 7*H*,*I*). However, 24 h later, *Tet1^FL^*-SID4X mice exhibited a reduction in their time spent freezing compared with *Tet1^FL^-*NFD controls (percent time freezing: *Tet1^FL^*-NFD, 41 ± 4.7%, *n* = 12 vs *Tet1^FL^*-SID4X, 26 ± 3.1%, *n* = 11, *t*_(21)_ = 2.6, *p* = 0.017; unpaired two-tailed *t* test), suggesting impaired memory (Fig. 7*J*). Moreover, *Tet1^S^*-SID4X mice exhibited a significant increase in freezing time compared with *Tet1^S^-*NFD controls (percent time freezing: *Tet1^S^*-NFD, 38 ± 2.6%, *n* = 11 vs *Tet1^S^*-SID4X, 55 ± 3.5%, *n* = 13, *t*_(22)_ = 3.8, *p* = 0.0010; unpaired two-tailed *t* test), indicative of a memory enhancement (Fig. 7*K*). Together, these data demonstrate that hippocampal-dependent memory formation is bidirectionally modulated by the neuron-specific actions of each *Tet1* isoform.

## Discussion

TET1 has been implicated in a wide variety of cognitive functions, most notably, learning and memory. However, the exact role of TET1 in the brain has remained ambiguous because of inconsistent findings reported between studies, suggesting key details regarding its function had yet to be elucidated. Here, we provide the first definitive evidence that two distinct isoforms of the *Tet1* gene are expressed in the adult mammalian brain. The first, *Tet1^FL^*, is transcribed at low basal levels in neurons and encodes for the full-length canonical TET1 enzyme, while the second, *Tet1^S^*, is the predominately expressed isoform in the brain, enriched in neurons, and encodes for a recently discovered enzyme variant that lacks a large portion of the N terminus, including the CXXC DNA binding domain (Zhang et al., 2016). Using isoform-specific genetic tools, we find that the individual disruption of *Tet1^FL^* and *Tet1^S^* in neurons has distinct effects on gene expression, excitatory synaptic transmission and memory formation, demonstrating that each isoform serves a non-redundant function in the nervous system.

In the first study to describe *Tet1^S^*, it was reported to be the only *Tet1* transcript expressed past early developmental stages (Zhang et al., 2016). However, others have shown in some adult tissues and/or cell types that both *Tet1* isoforms are co-expressed (Good et al., 2017; Yosefzon et al., 2017). We found that while *Tet1^S^* is the predominantly expressed isoform in the brain, *Tet1^FL^* is also actively transcribed. We attribute these inconsistencies to differences in the experimental techniques used to detect the lowly expressed *Tet1^FL^* transcript. For example, in the original report, a lack of *Tet1^FL^* expression was inferred using genome-wide RNA-seq datasets, while more recent studies, including our own, have used more sensitive methods such as cell-type specific ChIP-seq, RNAP2 ChIP-qPCR, 5′ RACE, and qRT-PCR.

We reported previously using immunohistochemistry that TET1 strongly co-localizes with the neuronal marker NeuN in the hippocampus, but only weakly with the astrocytic marker fibrillary acidic protein (GFAP), suggesting its enriched in neurons (Kaas et al., 2013). Our examination of *Tet1* isoform expression in neurons and glia supports these initial findings. For instance, while *Tet1^S^* is the most abundant transcript in both cell types, in relative terms, the short isoform is expressed at considerably higher levels in neurons (∼3-fold), while in glia, *Tet1^FL^* was ∼15-fold more abundant. Thus, the higher TET1 enrichment in neurons stems from greater *Tet1^S^* expression, whereas in glia, despite significantly higher levels of *Tet1^FL^*, exhibits lower overall expression because of reduced levels of the short transcript. These findings also suggest that the role of each isoform may be at least partially cell-type specific. In support of this idea, *Tet1^S^* transcript levels are downregulated in cultured neurons following depolarization and after fear learning in hippocampal CA1, whereas *Tet1^FL^*, which is much less abundant in excitable cells, remained at baseline levels. Similarly, greater relative expression of *Tet1^FL^* in glia may reflect its reported role as a tumor suppressor in gliomas (Fu et al., 2017), where its added presence might be necessary to control gene expression programs related to cellular proliferation.

Several studies using pan-*Tet1* KO mice have found that the loss of both isoforms provides cognitive benefits, including memory enhancement (Rudenko et al., 2013; Kumar et al., 2015; Feng et al., 2017; Cheng et al., 2018). Our findings that *Tet1^S^* repression alone is sufficient to enhance long-term fear memory suggests these pro-cognitive effects result from loss of the short isoform. These data are also consistent with our previous finding that acute overexpression of the TET1 catalytic domain, which also lacks the N-terminal domain of the full length enzyme, impairs memory formation (Kaas et al., 2013). Together, these observations strongly point to *Tet1^S^* as a memory suppressor, and perhaps more generally, as a negative regulator of neuroplasticity. In contrast, *Tet1^FL^* repression causes memory deficits. In agreement with these results, a recent study found that transgenic overexpression of *Tet1^FL^* causes enhanced memory formation and increased anxiety in mice (Kwon et al., 2018), indicating that overexpression of the full-length enzyme has the opposite effect on cognition. It is important to note that while our isoform-specific behavioral findings generally agree with previous reports, several groups have found that pan-*Tet1* deficient mice to either have normal memory or even exhibit an impairment (Zhang et al., 2013; Towers et al., 2018). The cause of these conflicting findings is still not clear. It seems likely, as previously posited (for review, see Alaghband et al., 2016; Antunes et al., 2019), that these differences reflect the use of different KO strategies in mice that target different exons and/or the presence of developmental confounds; the latter being particularly relevant as some *Tet1* mutant alleles display embryonic semi-lethality and/or smaller stature than littermates (Dawlaty et al., 2011; Kang et al., 2015; Towers et al., 2018).

Consistent with our behavioral data, individual disruption of *Tet1^FL^* and *Tet1^S^* in hippocampal neuron cultures had dissimilar effects on excitatory synaptic transmission. In particular, *Tet1^S^* depletion led to a significant reduction in mEPSC frequency. This presynaptically driven process has been shown to inversely correlate with the strength of long-term depression (LTD) in hippocampal slices (Zhang et al., 2005). Indeed, Rudenko et al. (2013) reported enhanced LTD in hippocampal slices from pan-*Tet1* KO mice with heightened memory retention. LTD has been shown to be necessary and sufficient to facilitate long-term spatial memory formation (Ge et al., 2010; Dong et al., 2012b), thus providing a rationale for how reduced mEPSC frequency might lead to enhanced memory in *Tet1^S^-*deficient mice. In contrast, transcriptional repression of *Tet1^FL^* caused both an increase in mEPSC frequency and amplitude. While more work is required to resolve why this is, our behavioral findings suggest that *Tet1^FL^* normally acts to suppress aberrant hyperexcitability that can lead to cognitive impairment.

Our transcriptomic data provides important insights into how *Tet1^FL^* and *Tet1^S^* repression alters neuronal physiology and cognition. For instance, loss of *Tet1^FL^* disrupted the expression of a large swath of the neuronal genome, causing a significant upregulation of cancer and immune response pathways as well as the downregulation of genes important for neuronal physiology and learning. These data point to the canonical isoform as a critical regulator of genomic stability in neurons and provides a straightforward explanation for the impaired memory in *Tet1^FL^-*deficient mice. Similarly, the hyperexcitability in *Tet1^FL^-*depleted neurons likely results, in part, from the induction of immune response genes. In particular, those associated with the tumor necrosis factor (Tnf) pathway, as its activation via the cytokine TNFα has been shown to be sufficient to increase excitability (Ming et al., 2013). Interestingly, previous transcriptomic analyses of constitutive pan-*Tet1* KO mice have not reported this activation of immune response genes (Rudenko et al., 2013; Zhang et al., 2013; Towers et al., 2018). We propose that the absence of an inflammatory response in these studies involves compensatory mechanisms during development, as viral-mediated conditional pan-*Tet1* KO in the nucleus accumbens has been shown to strongly induce immune gene expression (Feng et al., 2017). In the case of *Tet1^S^*, our transcriptomic data suggests that repression of the novel isoform may improve memory, by enhancing translation in neurons, as its loss resulted in the significant upregulation of genes encoding ribosomal RNAs (rRNAs) and proteins involved in ribosomal biogenesis. Consistent with this idea, a recent study has shown that learning-induced changes in rRNA expression are required for memory consolidation (Allen et al., 2018).

Although we do not address the molecular functions of the TET1^FL^ and TET1^S^ proteins in our study, recent findings provide some insights. For instance, the CXXC DNA binding domain-deficient TET1^S^ enzyme has been shown to exhibit lower chromatin affinity than TET1^FL^ when exogenously expressed in mESCs, yet still localizes to many of the same genomic features and gene targets as the canonical protein (Zhang et al., 2016). In addition, TET1^S^ expression resulted in a smaller increase in 5hmC than TET1^FL^, suggesting it might be a less potent, or more selective, methylcytosine dioxygenase. Both findings provide a rationale for the overlap we observed in DEGs between isoforms, and why *Tet1^S^* repression resulted in milder changes in gene expression than *Tet1^FL^*. Given that the CXXC domain directs TET1^FL^ to promoter-associated CpG islands (Xu et al., 2011), how TET1^S^ is recruited to its genomic targets remains an open question, but likely includes interactions with yet-to-be identified co-regulatory proteins. Recent published data suggests it might involve the transcription factors EGR1 (Sun et al., 2019) and FOXA1 (Yang et al., 2016), as blotting with a TET1 antibody after co-immunoprecipitation of these factors detected a band around 150 kDa, consistent with the predicted size of TET1^S^. Nevertheless, future studies addressing the molecular functions of *Tet1^S^* and *Tet1^FL^* will be needed to fully understand their roles in regulating nervous system function.

In conclusion, *Tet1^FL^* and *Tet1^S^* are co-expressed in the adult brain, and carry out distinct functions, providing important new insights into the role of TET enzymes in the nervous system. *Tet1^S^* repression enhances memory formation, suggesting that antagonists selective for the truncated enzyme may be an effective therapeutic strategy to treat cognitive deficits. *Tet1^FL^*, on the other hand, appears to be a critical regulator of neuroinflammation and cellular identity, suggestive of a role in aging, neurodegeneration and cancer. Overall, our results stress the importance of distinguishing between the two isoforms in future studies and provide the impetus to reexamine previous findings related to TET1 in depression, addiction and bipolar disorder (Dong et al., 2012a; Feng et al., 2015, 2017).
